# Unveiling the neuroprotective effects of pomegranate extract and/or physical activity against aluminum chloride-induced Alzheimer’s disease in rats: modulation of multitude pathways

**DOI:** 10.3389/fphar.2026.1793211

**Published:** 2026-05-19

**Authors:** Ahmed M. E. Hamdan, Hadil Alahdal, Haider F. S. Al-Zubaidy, Karema Abu-Elfotuh, Enji Reda, Yasmen F. Mahran, Asmahan Alghamdi, Heba Abdelnaser Aboelsoud, Samia A. Ismael, Ehsan K. Mohamed, Asmaa A. Amer, Jilan A. Nazeam, Alaadin E. El-Haddad, Khaled R. Abdelhakim, Magy R. Kozman, Noura M. Mohamed, Ayah M. H. Gowifel

**Affiliations:** 1 Department of Pharmacology and Toxicology, Faculty of Pharmacy, University of Tabuk, Tabuk, Saudi Arabia; 2 Prince Fahad Bin Sultan Chair for Biomedical Research (PFSCBR), Tabuk, Saudi Arabia; 3 Department of Biology, College of Science, Princess Nourah Bint Abdulrahman University, Riyadh, Saudi Arabia; 4 Department of Clinical Laboratory Sciences, Faculty of Pharmacy, University of Kufa, Najaf, Iraq; 5 Clinical Pharmacy Department, Faculty of Pharmacy (Girls), Al-Azhar University, Cairo, Egypt; 6 College of Pharmacy, Al-Ayen Iraqi University, AUIQ, An Nasiriyah, Iraq; 7 Department of Pharmacology and Toxicology, Faculty of Pharmacy, Sinai University – Kantara Branch, Ismailia, Egypt; 8 Department of Pharmacology and Toxicology, Faculty of Pharmacy, Ain Shams University, Cairo, Egypt; 9 Department of Basic Medical Sciences, College of Medicine, Prince Sattam Bin Abdulaziz University, Al-Kharj, Saudi Arabia; 10 Department of Anatomy and Embryology, Faculty of Medicine, Al-Azhar University, Cairo, Egypt; 11 Biochemistry Department Egyptian Drug Authority (EDA), Formerly National Organization of Drug Control and Research (NODCAR), Giza, Egypt; 12 Department of Pharmacognosy, Pharmaceutical and Drug Industries Research Institute, National Research Centre, Giza, Egypt; 13 Pharmacognosy Department, Faculty of Pharmacy, October 6 University, Giza, Egypt; 14 Histology Department, Misr University for Science and Technology, Cairo, Egypt; 15 Clinical Pharmacy Department, Faculty of Pharmacy, Misr University for Science and Technology, Giza, Egypt; 16 Department of Basic Sciences, College of Medicine, Princess Nourah Bint Abdulrahman University, Riyadh, Saudi Arabia; 17 Pharmacology and Toxicology Department, Faculty of Pharmacy, Modern University for Technology and Information (MTI), Cairo, Egypt

**Keywords:** Alzheimer’s disease, amyloidogenesis, autophagy, endoplasmic reticulum stress, P38 mitogen-activated protein kinase/c-jun N-terminal kinase/nuclear factor-kappa B/janus kinase 2/signal transducer and activator of transcription 3, physical activity, pomegranate extract

## Abstract

**Introduction:**

Alzheimer’s disease (AD) is a devastating neurodegenerative malady marked by cognitive dysfunction and increased deposition of amyloid-beta and hyperphosphorylated tau proteins. Aluminum-associated neurotoxicity is well-documented and manifests itself in the form of cognitive dysfunction. Nutraceuticals, such as pomegranate extract (POM) or physical activity (PHA), have been recognized to exert beneficial actions on human wellbeing. This study aimed to mechanistically scrutinize the effects of POM and PHA either separately or in combination on AlCl_3_-induced AD in rats.

**Methods:**

Sixty adult male Wistar rats were randomly assigned into five groups: control, AlCl_3_, AlCl_3_ + PHA, AlCl_3_ + POM, and AlCl_3_ + COMB (POM + PHA) and treated for 5 weeks.

**Results:**

PHA and POM, either alone or in combination, exhibited a significant boost in memory and cognitive abilities, evidenced by the significant improvements in behavioral outcomes, elevations in monoamine (DA, NE, 5-HT), LRP1, and neprilysin levels, and reductions in acetylcholinesterase (ACHE) activity, APOE4 levels, and AD markers, relative to the AlCl_3_-intoxicated group. All treatments significantly ameliorated AlCl_3_-induced perturbations in antioxidant, neurotrophic, autophagic, inflammatory, endoplasmic reticulum (ER) stress, and apoptotic parameters, along with histopathological and immunohistochemical findings. These effects were demonstrated by the significant increase in antioxidant (Nrf2/HO-1/TAC/SOD), neurotrophic (PI3K/AKT/CREB/BDNF/TrKB), and autophagic (AMPK/SIRT-1/Beclin-1) markers, with significant reduction in neuroinflammatory (P38 MAPK/JNK/NF-κB/JAK-2/STAT-3/TNF-α/IL-1β), ER stress (PERK/CHOP/GRP78), and apoptotic (BAX/P53/caspase-3) markers, relative to the AlCl_3_-intoxicated group. Interestingly, the combination exerted more favorable actions in all measured parameters and histological findings than the solo-treated groups.

**Conclusion:**

This study revealed the superiority of the combined therapy, compared to solo treatments, in alleviation of AlCl_3_-induced AD in rats, via adjustment of the Nrf2/HO-1, P38 MAPK/JNK/NF-κB/JAK-2/STAT-3, PI3K/AKT/GSK-3β/CREB, AMPK/SIRT-1, PERK/CHOP, and apoptotic hubs.

## Graphical Abstract

**Figure:**
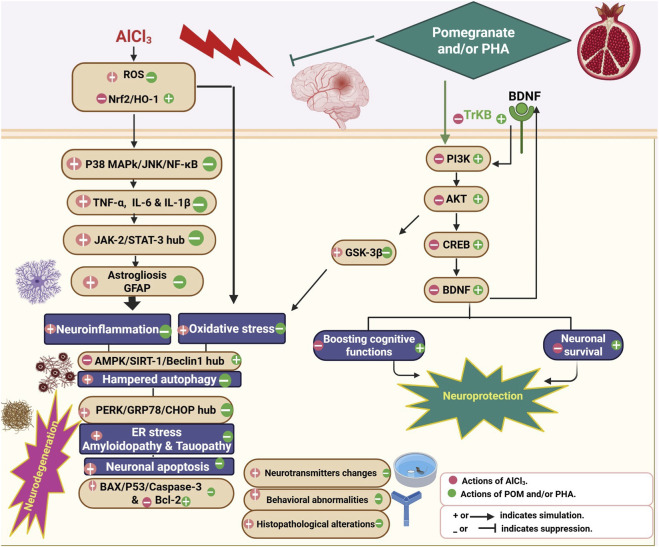

## Highlights

- POM and PHA, either alone or in combination, ameliorated AlCl_3_-induced biochemical, behavioral, and histopathological changes.
- The combination of POM with PHA exerted superior actions compared to standalone therapy.
- The combination ameliorated AlCl_3_-induced tauo-/amyloidopathy via modulation of the Nrf2/HO-1, MAPK/JNK/NF-κB, PI3K/AKT/CREB, AMPK/SIRT-1, PERK/CHOP, and amyloidogenic axes.
- The combined therapy may be considered a promising therapeutic approach for AD.

## 1 Introduction

Alzheimer’s disease (AD) is the most prevalent neurodegenerative disease, manifested by a progressive deterioration in cognitive and memory functions, along with behavioral abnormalities, lessening the quality of life for those individuals and posing serious socio-economic burdens (Vaz and Silvestre, 2020). AD is marked by perturbations in various neurotransmitter levels, comprising norepinephrine (NE), serotonin (5-HT), and dopamine (DA), along with a decrease in acetylcholine levels, owing to the excessive acetylcholinesterase (ACHE) levels, where these prominent cholinergic and monoaminergic dysfunctions strongly correlate with the decline in memory functions (Medhat et al., 2019).

AD is characterized by the build-up of abnormally folded proteins, particularly amyloid-beta (Aβ) and hyperphosphorylated tau protein (p-tau), forming amyloid plaques (Aβ plaques) and neurofibrillary tangles (NFTs), respectively (Khalifa et al., 2022). In this regard, amyloidogenesis is highlighted as the key pathological cascade in AD’s progression, in which the accumulation of Aβ, derived from the cleavage of the amyloid precursor protein (APP) by beta-secretase 1 (BACE1) and γ-secretase, results in the formation of insoluble Aβ plaques and NFTs of p-tau. Such neuronal alterations are accompanied by a decrease in memory and cognitive functions. Thus, AD is largely correlated with the prominent increase in the levels of the members of the amyloidogenic pathway, including APP, BACE1, and Aβ, along with the p-tau level, which are regarded as AD markers (Messiha et al., 2020). Of note, the increased levels of AD markers were reported to be tightly associated with the perturbation in redox status and heightened inflammatory response (Li et al., 2012; Alawdi et al., 2017). In addition, the higher levels of AD markers, particularly Aβ and p-tau, were reported to be firmly linked with the existence of an imbalance between their production and degradation/clearance, which is another pivotal feature in AD (Khodadadi et al., 2018).

In the context of the increased production, the level of apolipoprotein E4 (APOE4), which exists at elevated levels in AD and is regarded as a risk factor for AD, was linked with the increased amyloidogenesis, via promoting the expression of APP and BACE1. In parallel, the increased APOE4 level was stated to be associated with heightened tauopathy, through promoting kinases leading to the aberrant hyperphosphorylation of tau (Koutsodendris et al., 2022). In respect of the diminished degradation, the level of neprilysin, a metalloprotease enzyme responsible for the degradation of Aβ, had been noted as being reduced in AD (Khalifa et al., 2022). In the frame of the suppressed clearance, the expression of low-density lipoprotein receptor-related protein 1 (LRP1), which is a receptor for transporting Aβ to the circulation, serving as a sink by clearing it from the brain, has been reported to be diminished in AD (Xu et al., 2023). Ample evidence has shown that reduced neprilysin and LRP1 levels are strictly linked with the increased Aβ and p-tau levels in AD (Khalifa et al., 2020; Liu et al., 2024; Yang et al., 2025).

The pathophysiology of AD is not confined to amyloidogenesis solely. AD has a multifactorial origin involving myriad interconnected pathological events and molecular pathways (Messiha et al., 2020). In addition, the role of increased oxidative stress and neuroinflammation has been underscored in the pathophysiological process of neurodegeneration, where these two pathomechanisms are tightly interrelated (Rather et al., 2018). Undeniably, oxidative stress is a cardinal event in the etiopathology of neurodegenerative ailments (Angelova and Abramov, 2018). Because the brain is rich in polyunsaturated fatty acids (PUFAs), it is highly vulnerable to the deleterious actions of reactive oxygen species (ROS) with the depletion in the antioxidant defense machineries and the consequent fostering of both neurodegeneration and neuronal apoptosis (Javed et al., 2012). Among the pivotal axes in charge of the regulation of antioxidant response is the nuclear factor-erythroid 2-related factor 2 (Nrf2) hub, which controls the expression of various antioxidant genes, including heme oxygenase-1 (HO-1), thereby eliminating ROS (Awad et al., 2023).

In addition to the overstated oxidative stress, the increased inflammatory response has been highlighted as a key pathophysiological event in neurodegenerative maladies (Javed et al., 2012). One of the prime players in the inflammatory response is the P38 mitogen-activated protein kinase (P38 MAPK)/c-Jun N-terminal kinase (JNK) axis, which was stated to be provoked by excessive ROS levels (Saha et al., 2020; Hosseini et al., 2024). Once the P38 MAPK/JNK axis is activated, it activates the nuclear factor-kappa B (NF-κB) hub, a key player in inflammatory response, leading to the generation of pro-inflammatory mediators, including tumor necrosis factor-alpha (TNF-α), interleukin-6 (IL-6), and IL-1β, that eventually trigger neuronal damage and neurodegeneration (Yang et al., 2014; Kim et al., 2021). Another contributor implicated in neuroinflammation is the Janus kinase-2 (JAK-2)/signal transducer and the transcription-3 (STAT-3) hub that, when activated by pro-inflammatory mediators, initiates the phosphorylation of STAT-3, resulting in astroglial activation, amplification of inflammatory response, and neuronal apoptosis (Jain et al., 2021). Based upon the notion that neuroinflammation and oxidative stress are two interlocked pathological events in neurological maladies, the activation of the Nrf2/HO-1 hub can inhibit P38 MAPK/JNK/NF-κB activation via elimination of ROS, resulting in curbing both oxidative stress and neuroinflammation (Huang C. et al., 2018; Qi et al., 2019). In this respect, Nrf2 enhancers may be regarded as promising strategies for the amelioration of neurological disorders, via dampening both ROS levels and inflammatory pathways.

Another event that plays a crucial part in the pathophysiology of neurodegenerative maladies is the dysregulation of phosphoinositide 3-kinase (PI3K)/protein kinase B (AKT) signaling, which exacerbates oxidative stress, neuroinflammation, and neuronal apoptosis (Beilharz et al., 2014; You et al., 2020). Activation of the PI3K/AKT axis counteracts redox imbalance, neuroinflammation, and apoptosis, via suppressing glycogen synthase kinase-3-beta (GSK-3β) and increasing cyclic adenosine monophosphate (cAMP) response element-binding protein (CREB) (Luo et al., 2021). In addition, CREB activation promotes the generation of brain-derived neurotrophic factor (BDNF), which stimulates tropomyosin receptor kinase B (TrkB), thereby boosting neuronal survival, neuronal plasticity, and memory functions (Bagheri et al., 2021). In this regard, modulation of the PI3K/AKT/GSK-3β/CREB cue may be considered an attractive therapeutic target for alleviation of neurodegenerative disorders, including AD.

Protein homeostasis, proteostasis, is vital for maintaining neuronal health, where it acts through controlling protein synthesis, folding, and the eradication of misfolded proteins. Hence, dysregulation of proteostasis is a pivotal contributor in the pathophysiology of neurodegenerative maladies, where it is associated with the incidence of proteinopathy, an important feature in neurodegenerative disorders, including AD (Höhn et al., 2020). Autophagy is a key player in maintaining proteostasis, and its impairment is a prominent feature in the etiopathology of neurodegenerative maladies (Gu et al., 2021; Barmaki et al., 2023). Autophagy is an indispensable process for the selective eradication of misfolded proteins and damaged organelles, thereby maintaining neuronal survival and cognitive function. Reduced autophagy is firmly linked with the excessive deposition of misfolded proteins, proteinopathy, and neuronal damage (Fowler and Moussa, 2018).

AMP-activated protein kinase (AMPK)/sirtuin-1 (SIRT-1) signaling is the primary axis in charge of regulating the autophagic processes, and the stimulation of both SIRT-1 and AMPK increases autophagy by boosting the expression of autophagy-related markers, specifically Beclin 1. p62 is a ubiquitin-binding protein that selectively detects autophagic cargo to mediate its engulfment into autophagosomes. p62 accumulates when autophagy is reduced and *vice versa*. Hence, it serves as a negative indicator for autophagy (Qu et al., 2019; Deng et al., 2020). Activation of the AMPK/SIRT-1 axis increases autophagy, suppresses the accumulation of misfolded proteins, inhibits neuronal apoptosis, and ameliorates neurodegeneration (Garcia and Shaw, 2017). Therefore, the modulation of the AMPK/SIRT-1 axis is a promising approach for mitigating neurodegeneration and cognitive dysfunction.

In addition to the defective autophagy, excessive endoplasmic reticulum (ER) stress may play a fundamental part in the etiopathology of neurodegenerative illnesses, via promoting neuronal apoptosis (Ghemrawi and Khair, 2020). Pathogenic and stressful conditions, including increased oxidative stress and inflammation, and diminished autophagy, were reported to be closely linked with unusual protein misfolding and accumulation inside the ER, proteinopathy, and the incidence of heightened ER stress (Hetz et al., 2020). It is important to mention that ER stress instigates the unfolded protein response (UPR) in order to relieve ER stress or otherwise induce apoptosis (Zhang et al., 2018). Among UPR sensors is protein kinase RNA-like ER kinase (PERK), which is activated during ER stress via liberation from glucose-regulated protein 78 (GRP78) (Zhang et al., 2020). Under prolonged ER stress, the UPR cascade fails to restore ER homeostasis. The hyperactivation of UPR shifts toward promoting apoptosis, through stimulating the expression of the pro-apoptotic C/EBP homologous protein (CHOP). Thus, increased ER stress is strongly related to the occurrence of neuronal apoptosis (Huang H. et al., 2018). In this way, reducing ER stress could inhibit neuronal apoptosis and promote neuronal survival.

Exposure to heavy metals has been associated with prominent neurotoxic effects (Raj et al., 2021). Among the most famous heavy metals with reported neurotoxic effects is aluminum (Al). Al is widely included in various daily necessities, such as drinking water, aluminum utensils, foil paper, and food packaging, which greatly enlarges the risk of exposure to Al in humans (Al-Hashem, 2009). Long-term exposure to Al is accompanied by its accumulation in the brain, eliciting its neurotoxic effects through triggering oxidative stress, neuroinflammation, and neuronal apoptosis, along with alterations in neurotransmitter levels, neurodegeneration, and cognitive dysfunction (Rather et al., 2019). Al provokes hyperphosphorylation of tau, which, together with promoting the build-up of Aβ, has been recognized as a hallmark for AD (Zhang et al., 2019; Kaur et al., 2021).

To date, available treatments for AD, including ACHE inhibitors, such as donepezil, and NMDA receptor antagonists, such as memantine, simply alleviate the symptoms while exhibiting side effects over long-term use (Cummings et al., 2014; Liu et al., 2018). Hence, there is an urgent need to focus on finding safer alternative approaches for the mitigation of AD. The use of natural products and plant extracts, possessing neuroprotective potentials, has garnered remarkable attention, owing to their well-known safety profiles, along with their multi-pharmacological actions, including antioxidant and anti-inflammatory activities, making them promising candidates for the amelioration of AD (Ratheesh et al., 2017; Raj et al., 2021).

Pomegranate (*Punica granatum* L.), which is broadly utilized in folk medicine and is considered a functional food, exhibits a wide range of health benefits (Johanningsmeier and Harris, 2011). It contains numerous groups of phytochemicals, chiefly phenolic compounds, including flavonoids (anthocyanins, catechins, and other complex flavonoids) and hydrolyzable tannins (ellagic acid and punicalagin) (Ismail et al., 2012; Abozid and Sci, 2013). These bioactive constituents possess various beneficial properties, including antioxidant, anti-inflammatory, anticancer, neuroprotective, and anti-apoptotic actions (Guo et al., 2007; Doostan et al., 2017; Alami et al., 2024). Notably, pomegranate extract (POM) was reported to exhibit superior actions compared to those of its single constituents, possibly due to the synergistic effects between its components (Ahmed et al., 2014). Previous studies outlined the advantageous effects of POM against various chronic diseases, such as diabetes, cancer, and neurological disorders (Amri et al., 2017; Fathy et al., 2021).

Another attractive approach is physical activity (PHA), which has been demonstrated to possess favorable actions against several diseases, specifically neurological conditions. Interestingly, PHA was reported to attenuate various pathophysiologic mechanisms that are directly linked with the incidence of neurological maladies. For example, PHA could inhibit the inflammatory response, improve antioxidant status, and enhance memory and cognitive functions while safeguarding against neuronal damage (Lu et al., 2017; Rosa et al., 2021). Therefore, PHA may be regarded as a strategy for the amelioration of neurodegenerative disorders. Because the pathophysiology of neurodegenerative diseases, including AD, is complicated, and various interrelated pathways are implicated (Awad et al., 2023), the use of a combination regimen that targets numerous cascades has become a promising approach.

We hypothesized that the use of a multimodal combination regimen that integrates PHA and the nutraceutical POM may possess beneficial effects against AD. Accordingly, the aims of our study were to first, scrutinize and compare the neuroprotective effects of POM, PHA, and their combination against AlCl_3_-induced AD in rats. Second, our aims were extended to offer mechanistic insights into the molecular targets through which these agents exert their favorable actions, focusing on their possible modulatory effects on Nrf2/HO-1, P38 MAPK/JNK/NF-κB, JAK-2/STAT-3, PI3K/AKT/GSK-3β/CREB, AMPK/SIRT-1, PERK/CHOP, and amyloidogenic and apoptotic axes, as probable contributors to AD’s pathophysiology.

## 2 Materials and methods

### 2.1 Materials

#### 2.1.1 Plant material

Pomegranate extract (POM) was obtained from Gongyi Xiangrui Eco Material Co. Ltd (China). POM was freshly prepared in 1% Tween 80 in distilled water (Battineni et al., 2017) and administered to rats at a dose of 250 mg/kg, orally, daily (Ahmed et al., 2014; Hashem et al., 2017; Alshinnawy et al., 2020) for 5 weeks.

#### 2.1.2 Chemicals

Aluminum chloride (AlCl_3_) (CAS Number 7446-70-0) was acquired from Sigma-Aldrich Co. Inc. (St. Louis, MO, United States). AlCl_3_ was freshly prepared in normal saline (0.9% sodium chloride solution) and administered to rats at a dose of 70 mg/kg, intraperitoneally, daily, for 5 weeks to induce neurotoxicity (Omran et al., 2022; Elariny et al., 2024). All chemicals and solvents employed in the study were commercially available and of the highest grade.

#### 2.1.3 Animals and ethical statement

Sixty adult male Wistar rats, weighing between 220 g and 250 g, were obtained from Nile Co. for Pharmaceuticals and Chemical Industries, Cairo, Egypt. The rats were acclimatized for 1 week before the start of the experiments. The rats were housed at the animal house facility in the Faculty of Pharmacy for Girls, Al-Azhar University, in stainless steel cages, under controlled conditions (temperature of 25 ± 1 °C, humidity of 50% ± 5%, with 12-h light and dark cycles). The animals had access to a normal dietary pellet and water *ad libitum*. All experimental procedures were agreed and supervised by the Animal Care and Use Committee of the Faculty of Pharmacy, Al-Azhar University, with ethical approval number 356/2022. The handling of animals was in accordance with the Animal Research: Reporting of In Vivo Experiments (ARRIVE) recommendations and the guidelines documented in “Guide for Care and Use of Laboratory Animals,” published by the National Institutes of Health (NIH Publications No. 8023, revised 1978).

### 2.2 Methods

#### 2.2.1 Phytochemical studies

##### 2.2.1.1 POM fractionation

POM powder (500 mg) was fractionated in Soxhlet with methanol (80%, 2 × 50 mL). The filtered extract was evaporated (Rotavapor®, BÜCHI, Switzerland) and was used for chemical investigations.

##### 2.2.1.2 UPLC–ESI–Q-TOF–MS/MS analysis and metabolite identification

The analysis of the sample was performed via liquid chromatography–electrospray ionization–tandem mass spectrometry (LC–ESI–MS/MS) using an ExionLC AC system for separation and SCIEX Triple Quad 5500+ MS/MS system equipped with electrospray ionization (ESI) for detection. The separation was performed using an Ascentis® C18 Column (4.6 × 150 mm, 3 µm). The mobile phase consisted of two eluents, A: 0.1% formic acid and B: acetonitrile (LC grade). The mobile phase gradient was programmed as follows: 10% B at 0–1 min, 10%–90% B from 1 min to 33 min, 90% B from 33 min to 37 min, 10% B at 37.1 min, and 10% B from 37.1 min to 40 min. The flow rate was 0.7 mL/min, and the injection volume was 10 µL. For MS/MS analysis, negative ionization mode was applied with a scan (EMS-IDA-EPI) from 100 Da to 1,000 Da for MS1 with the following parameters: curtain gas: 25 psi; IonSpray voltage: −4,500 eV; source temperature: 500 °C; ion source gas 1 and 2: 45 psi; mass scan range: 50 Da to 800 Da for MS2; with a declustering potential: −80; collision energy: −35; collision energy spread: 15. Compound identification was performed using MS-DIAL software version 4.70 with Fiehn HILIC as an identification library for alignment. Characterization of metabolites was performed using their UV–VIS spectra (220–600 nm) and exact masses, in addition to MS2 in both ionization modes, Rt data, reference literature, and searching the phytochemical dictionary of natural products.

#### 2.2.2 Experimental design

##### 2.2.2.1 Animal grouping

Following the 1-week acclimatization period, rats were randomly allocated into five groups of 12 rats per group, in the following way.

- Group I (normal control group): rats were intraperitoneally injected with normal saline (1 mL/kg/day) and orally administered with 1% Tween 80 in distilled water (1 mL/kg/day) for 5 weeks.
- Groups II–V: animals were injected with AlCl_3_ (70 mg/kg/day), intraperitoneally (Omran et al., 2022; Elariny et al., 2024), and treated for 5 weeks as follows:
- Group II (AlCl_3_ group): animals received 1% Tween 80 in distilled water (1 mL/kg/day), orally, for 5 weeks.
- Group III (AlCl_3_ + PHA): rats were exposed to physical activity (PHA), swimming for 60 min/day, 5 days/week, for 5 weeks.
- Group IV (AlCl_3_ + POM): rats were orally administered with pomegranate extract (POM) (250 mg/kg) (Ahmed et al., 2014; Hashem et al., 2017; Alshinnawy et al., 2020), daily, for 5 weeks.
- Group V (AlCl_3_ + COMB): rats were treated with POM (250 mg/kg), orally, daily, and also exposed to PHA in the form of swimming for 60 min/day, 5 days/week, for 5 weeks.

Behavior tests were performed 24 h after the end of the experimental period. Animals were anesthetized with an intraperitoneal injection of a ketamine (75 mg/kg, i.p.) and xylazine (10 mg/kg, i.p.) mixture (El-Shoura et al., 2023; Khidr et al., 2023) and euthanized by decapitation (Hassan et al., 2024; Gad et al., 2025) 24 h after the termination of behavioral tests. Then, the brains were promptly removed for biochemical analyses and histopathological evaluations.

##### 2.2.2.2 Physical activity

Physical activity (PHA) was employed as a therapeutic strategy to inhibit neurotoxicity induced by AlCl_3_. PHA was performed by exposing the animals to a swimming exercise protocol comprising 60 min/day, 5 days/week, for 5 weeks.

###### 2.2.2.2.1 Swimming exercise protocol

The following swimming exercise protocol was in accordance with the methods of Viboolvorakul and Patumraj (2014) and Muhammad et al. (2024), who reported that this swimming protocol exerted neuroprotective and cognitive-enhancing actions. The swimming exercise protocol comprises adaptation of animals to swimming in a cylindrical tank (80 cm diameter and 90 cm depth), filled with 60 cm of water that was maintained thermostatically at a temperature of 33 °C–36 °C. The swimming exercise protocol involved training once daily for 15 min for 2 days, then the duration was gradually increased by an additional 15 min daily, until the animals swam for 60 min on the fifth day of the first week. Subsequently, the swimming exercise protocol was continued with a swimming period of 60 min/day, 5 days/week, for 5 weeks. The procedure was carried out between 10:00 a.m. and 12:00 p.m. At the end of each exercise session, the rats were towel-dried and returned to their cages. A rest for 24 h was permitted before rats were subjected to the behavioral tests.

#### 2.2.3 Behavioral tests

The following behavioral tests were done.

##### 2.2.3.1 Y-maze test

The Y-maze test, in accordance with the documented protocol by Hritcu et al. (2012), was utilized to assess the spatial working memory by calculating the spontaneous alteration percentage (SAP). As previously reported, a black wooden Y-maze with three arms (35 cm long, 25 cm tall, and 10 cm wide) labeled A, B, or C and a symmetrical triangular central area was utilized (Sarter et al., 1988). Animals were placed at the edge of one arm and permitted to roam freely over the maze for 8 min. The entries were counted when the rat’s hind paws were entirely inside the arm. The number of entries into each arm and the rats' natural tendency to alternate between the three arms were recorded, respectively. SAP was calculated using the following formula: [number of alternations/(total arm entries − 2)] × 100. Prior to testing the next rat, the maze was washed with an ethanol solution and dried with a towel to eliminate any lingering scents.

##### 2.2.3.2 Morris water maze test

Spatial learning and memory retention in rats were examined using the Morris water maze (MWM) test (Morris, 1984; Sharma, 2009; Bromley-Brits et al., 2011). Tap water, 25 °C ± 2 °C, was poured into a circular water tank measuring 150 cm in diameter and 60 cm in height to a depth of 30 cm. The pool was virtually divided into four equal quadrants (east, west, north, and south). For the acquisition test, an escape platform measuring 10 cm in diameter was submerged 2 cm beneath the water’s surface at a fixed place in the center of one quadrant. During the trial, the platform remained in the same quadrant. The rats were first given four acquisition days with four 60-s trials per day, during which each rat was positioned into the water with its back toward the pool wall from a specific site in each quadrant and allowed to swim to the platform. The animals had a maximum of 60 s to locate the platform before being allowed to rest on it for 20 s prior to the beginning of the next trial. If it took more than 60 s to find the platform, the rat was gently given 20 s to rest. The escape latency, which is the rat’s average time to find the platform, was recorded. On the fifth day, a probe test was done by allowing the animals to swim freely for 60 s while removing the platform and measuring the time spent by each animal in the nominated quadrant.

#### 2.2.4 Tissue sampling and preparation

Subsequent to euthanasia, the animals’ brains were removed and washed with ice-cold saline. Six brains from each group were fixed in 10% neutral buffered formalin overnight for subsequent histopathological and immunohistochemical investigations. Each of the remaining six brains in each group was divided into two equal parts. The first part was separately homogenized in ice-cold PBS (pH = 7.4) to obtain 10% homogenate (w/v). Subsequently, the homogenate was centrifuged at 1800 g for 10 min at 4 °C, and the obtained supernatant was utilized for various biochemical analyses, including enzyme-linked immunosorbent assay (ELISA). The second part was kept at −80 °C to be utilized in real-time polymerase chain reaction (PCR) analyses.

#### 2.2.5 Biochemical assessments

##### 2.2.5.1 Colorimetric analyses

The levels of superoxide dismutase (SOD), malondialdehyde (MDA), and total antioxidant capacity (TAC) were measured according to the methods delineated by Nishikimi et al. (1972), Ohkawa et al. (1979), and Koracevic et al. (2001), respectively. All these colorimetric assessments were accomplished on the supernatant of 10% brain tissue homogenate by using assay kits acquired from Biodiagnostic, Inc., Giza, Egypt, according to the manufacturer’s instructions.

##### 2.2.5.2 Enzyme-linked immunosorbent assay in brain tissues

The concentrations of numerous biomarkers, including BACE1, Aβ, p-tau, APOE4, neprilysin, TNF-α, IL-1β, IL-6, BDNF, ACHE, and caspase-3, were assessed using enzyme-linked immunosorbent assay (ELISA) kits provided by MyBioSource, Inc. (San Diego, United States). The measurements were performed on the supernatant of 10% brain tissue homogenate, in accordance with the manufacturer’s guidelines.

##### 2.2.5.3 Fluorometric assays in the brain

Brain monoamine neurotransmitters, including DA, NE, and 5-HT, were measured instantaneously, in accordance with Ciarlone (Ciarlone, 1978). As previously documented, monoamine neurotransmitters were detected fluorometrically within samples at λ_ex_/λ_em_ 320/480 nm, 380/480 nm, and 355/470 nm for DA, NE, and 5-HT, respectively (Ciarlone, 1978).

##### 2.2.5.4 Analysis of gene expression by real-time quantitative polymerase chain reaction

Real-time quantitative polymerase chain reaction (RT‒qPCR) was performed using Applied Biosystems Step One Plus instruments to assess the mRNA levels of PI3K, AKT, GSK-3β, CREB, TrKB, Nrf2, HO-1, APP, LRP1, NF-κB, P38 MAPK, JNK, PERK, CHOP, GRP78, JAK-2, STAT-3, AMPK, SIRT-1, Beclin-1, P62, P53, B-cell lymphoma 2 protein (Bcl-2), B-cell lymphoma protein 2 (Bcl-2)-associated X protein (BAX), and the housekeeping gene (β-actin) in the brain. Total RNA was isolated using a QIAGEN Tissue Extraction Kit (QIAGEN, Germantown, MD, United States), in accordance with the manufacturer’s guidelines. Reverse transcription of the extracted mRNA was done utilizing the SensiFAST™ cDNA Synthesis Kit (Cat No. BIO-65053). The samples were normalized to β-actin expression. The relative expression of the target genes (PI3K, AKT, GSK-3β, CREB, TrKB, Nrf2, HO-1, APP, LRP1, NF-κB, P38 MAPK, JNK, PERK, CHOP, GRP78, JAK-2, STAT-3, AMPK, SIRT-1, Beclin-1, P62, P53, Bcl-2, BAX, and β-actin) was calculated using the following formula: 2^^(−ΔΔCT)^ (Livak and Schmittgen, 2001). The forward and reverse sequences of the primers used for PCR amplification are shown in Table 1.

**TABLE 1:** Primer sequences utilized for RT-qPCR analysis.

Gene	Forward and backward sequences
*PI3K*	F: 5′-GCCCAGGCTTACTACAGAC-3′ R: 5′-AAGTAGGGAGGCATCTCG-3′
*AKT*	F: 5′-ATGGACTTCCGGTCAGGTTCA-3′ R: 5′-GCCCTTGCCCAGTAGCTTCA-3′
*GSK-3β*	F: 5′-TCGCCACTCGAGTAGAAGAAA-3′ R: 5′-ACTTTGTGACTCAGGAGAACT-3′
*CREB*	F: 5′-CAGACAACCAGCAGAGTGGA-3′ R: 5′-CTGGACTGTCTGCCCATTG-3′
*TrkB*	F: 5′-CCTCCACGGATGTTGCTGA-3′ R: 5′-GGCTGTTGGTGATACCGAAGTA-3′
*AMPK*	F: 5′-AAAGAACCCTAGCCTGAAGAGG-3′ R: 5′-ACCTTCCGAGATGAATGCTTTT-3′
*SIRT-1*	F: 5′-GGCACCGATCCTCGAACAAT-3′ R: 5′-CGCTTTGGTGGTTCTGAAAGG-3′
*Beclin-1*	F: 5′-AGCACGCCATGTATAGCAAAGA-3′ R: 5′-GGAAGAGGGAAAGGACAGCAT-3′
*P62*	F: 5′-GGAAGCTGAAACATGGGCAC-3′ R: 5′-CCAAGGGTCCACCTGAACAA-3′
*Nrf2*	F: 5′-CTCTCTGGAGACGGCCATGACT-3′ R: 5′-CTGGGCTGGGGACAGTGGTAGT-3′
*HO-1*	F: 5′-CACCAGCCACACAGCACTAC-3′ R: 5′-CACCCACCCCTCAAAAGACA-3′
*APP*	F: 5′-GGATGCGGAGTTCGGACATG-3′ R: 5′-GTTCTGCATCTGCTCAAAG-3′
*LRP1*	F: 5′-CAAGATGTATGAAGGTGGAGAGC-3′ R: 5′-ACTGGGTTGGTGAAGTTGGTAG-3′
*P38 MAPK*	F:5′-ATAATGCGTCTGACGGGGAC-3′ R:5′-GGGTCGTGGTACTGAGCAAA-3′
*JNK*	F:5′-AGTGTAGAGTGGATGCATGA-3′ R:5′-ATGTGCTTCCTGTGGTTTAC-3′
*BAX*	F: 5′-CACGTCTGCGGGGAGTCA-3′ R: 5′-TAGGAAAGGAGGCCATCCCA-3′
*Bcl-2*	F: 5′-GGATGACTTCTCTCGTCGCTAC-3′ R: 5′-TGACATCTCCCTGTTGACGCT-3′
*P53*	F: 5′-GTCGGCTCCGACTATACCACTATC-3′ R: 5′-CTCTCTTTGCACTCCCTGGGGG-3′
*PERK*	F: 5′-GCCGATGGGATAGTGATG-3′ R: 5′-GCAGCCTCTACAATGTCTTCT-3′
*CHOP*	F: 5′-TCTGCCTTTCGCCTTTGAG-3′ R: 5′-GCTTTGGGAGGTGCTTGTG-3′
*GRP78*	F:5′-TAATCAGCCCACCGTAAC-3′ R:5′-GTTTCCTGTCCCTTTGTC-3′
*JAK-2*	F: 5′-AGCTCCTCTCCTTGACGACT-3′ R:5′-GCACGCACTTCGGTAAGAAC-3′
*STAT-3*	F: 5′-CAAAGAAAACATGGCCGGCA-3′ R:5′-GGGGGCTTTGTGCTTAGGAT-3′
*NF-κB*	F: 5′-CGCGGGGACTATGACTTGAA-3′ R:5′-AGTTCCGGTTTACTCGGCAG-3′
*β-actin*	F: 5′-CCGTAAAGACCTCTATGCCA-3′ R: 5′-AAGAAAGGGTGTAAAACGCA-3′

#### 2.2.6 Histopathological and immunohistochemical examinations

##### 2.2.6.1 Histopathological examinations

Brain tissue samples from various groups were fixed in 10% formalin for 1 day before being rinsed with tap water. Dehydration was carried out using an ascending sequence of ethyl alcohol. Clearing and paraffin embedding were performed in a hot air oven at 56 °C for 1 day. For routine microscopic examination with a light microscope, 5 µm sections were mounted on glass slides, deparaffinized, and stained with hematoxylin and eosin (H&E), and then photomicrographs at a magnification of ×40 were captured (Bancroft and Layton, 2013).

##### 2.2.6.2 Immunohistochemical examinations

Subsequent to sectioning and deparaffinization of the blocks of brain tissues by xylene, samples were rehydrated using descending concentrations of alcohol. Afterward, the sections were kept in 3% H_2_O_2_ for 10 min, then in 0.1% trypsin for 30 min, at 37 °C, for antigen retrieval. Next, sections from each group were incubated overnight at 4 °C with monoclonal mouse antibodies against glial fibrillary acidic protein (GFAP) (1:800 dilution; Servicebio, Cat# GB12090). The sections were treated with the secondary antibody, biotinylated goat anti-rabbit (Invitrogen), stained with 3, 3-diaminobenzidine, at 37 °C for 30 min, and ultimately counterstained with hematoxylin. Finally, image analysis was performed by assessing the area percent (A %) in 10 microscopic fields using ImageJ software (version 1.48).

#### 2.2.7 Statistical analysis

Data were represented as the mean ± SEM. Multiple comparisons were performed using one-way ANOVA, followed by Tukey’s multiple comparison test as a *post hoc* test to evaluate group variation. Two-way ANOVA was used to assess the statistical analysis of the escape latency time for 4 days in the MWM test. GraphPad Prism Software Ver. 5 (ISI®, United States) was employed to perform all statistical analyses and data visualization. The level of significance was considered to be P <0.05.

## 3 Results

### 3.1 Phytochemical screening

#### 3.1.1 UPLC–ESI–Q-TOF–MS/MS analysis and metabolite identification

**FIGURE 1:**
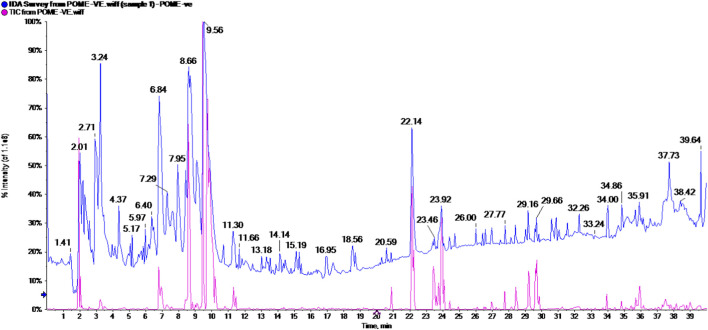
Total ions chromatogram (TIC) of POM in negative ionization modes.

The total ion chromatogram (TIC) in negative mode is presented in Figure 1 and Table 2. Metabolite assignments were made by comparing retention time, UV/VIS, and MS/MS data of detected compounds, whenever available, or interpreting MS data combined with chemo-taxonomic data reported in the literature or databases.

**TABLE 2:** Metabolites tentatively identified via UPLC–ESI–Q-TOF–MS/MS in the plant extract using negative ionization modes.

#	Rt	Proposed compound	Formula	[M-H] m/z	MS2 (characteristic fragments)
Sugar derivatives					
1	1.91	Gluconic acid	C_6_H_12_O_7_	195.10	128, 159, 177
2	2.01	Arabinose	C_5_H_10_O_5_	149.05	--
3	3.09	Arabitol	C_5_H_12_O_5_	151.10	--
Organic acids					
4	1.51	Fumaric acid	C_4_H_4_O_4_	115.02	68
5	2.01	Tartaric acid	C_4_H_5_O_6_	148.90	--
6	2.01	Quinic acid	C_7_H_12_O_6_	191.15	155, 96, 59
7	7.29	Ascorbic acid	C_6_H_8_O_6_	175.01	--
8	7.97	Malic acid	C_4_H_6_O_5_	132.85	115, 89, 71
Phenolics and flavonoid derivatives					
9	3.02	Kaempferol-O-rutinoside	C_27_H_30_O_15_	593.25	285
10	3.03	Isorhamnetin-3-O-rutinoside	C_28_H_32_O_16_	623.1	315, 299
11	5.82	Protocatechuic acid	C_7_H_6_O_4_	153.03	109
12	6.42	Hydroxycinnamic acid	C_9_H_8_O_3_	163.05	103
13	7.29	Dihydroxymandelic acid	C_8_H_8_O_5_	182.95	--
14	7.97	Shikimic acid	C_7_H_10_O_5_	173.05	155
15	9.33	Myricetin	C_15_H_10_O_8_	317.1	137, 125, 109
16	11.1	Methoxycatechol	C_7_H_8_O_3_	139.04	111
17	14.65	Caffeic acid hexoside	C_15_H_18_O_9_	341.30	179, 161, 135
18	11.65	Isorhamnetin	C_16_H_11_O_7_	315.1	300, 151, 137, 125
19	14.86	Coumaroyl hexoside	C_15_H_18_O_8_	325.30	183, 117
20	16.48	Kaempferol	C_15_H_10_O_6_	285.2	137, 125, 109
Tannins					
21	9.75	Ellagic acid	C_14_H_6_O_8_	301.01	229, 185
22	10.98	Epigallocatechin	C_15_H_14_O_7_	305.27	275, 125
24	11.96	Catechin/epicatechin	C_15_H_14_O_6_	289.07	261, 245, 179
25	21.7	Galloyl-hexoside	C_13_H_16_O_10_	331	169, 271
26	22.12	Punicalagin (HHDP-gallagyl-hexoside)	C_48_H_28_O_30_	1,083.62	541
Anthocyanins					
27	2.28	Pelargonidin	C_15_H_11_O_5_^+^	271.33	181
28	13.69	Cyanidin chloride	C_15_H_11_ClO_6_	321.63	285
29	15.29	Delphinidin-3-hexoside	C_21_H_21_O_12_	463.01	301
30	23.94	Pelargonidin-3-hexoside chloride	C_21_H_21_ClO_10_	467.29	437
31	36.06	Cyanidin-3-*O*-(6-acetyl-hexoside)	C_23_H_22_O_12_	489.25	447, 285
Fatty acids					
32	3.55	Hydroxymyristic acid	C_14_H_28_O_3_	243.20	225, 207
33	6.85	Palmitoleic acid	C_16_H_30_O_2_	253.35	--
34	9.02	Hydroxydecanoic acid	C_10_H_20_O_3_	187.10	187,159, 127, 143
35	9.57	Palmitic acid	C_16_H_32_O_2_	255.25	--
36	9.87	Linoleic acid	C_18_H_32_O_2_	279.35	--
37	12.79	Octadecanedioic acid	C_18_H_34_O_4_	313.20	298, 270
38	14.94	Heneicosanoic acid	C_21_H_42_O_2_	325.20	283, 265
39	16.81	Docosatetraenoic acid	C_22_H_36_O_2_	331.45	--
40	17.35	Docosahexanoic acid	C_22_H_32_O_2_	327.30	283, 191, 229

Diff. (ppm) was below 10 ppm.

### 3.2 Experimental results

#### 3.2.1 Effects of PHA and POM, either alone or in combination, on AlCl_3_-induced behavioral alterations in the Y-maze and MWM tests

**FIGURE 2:**
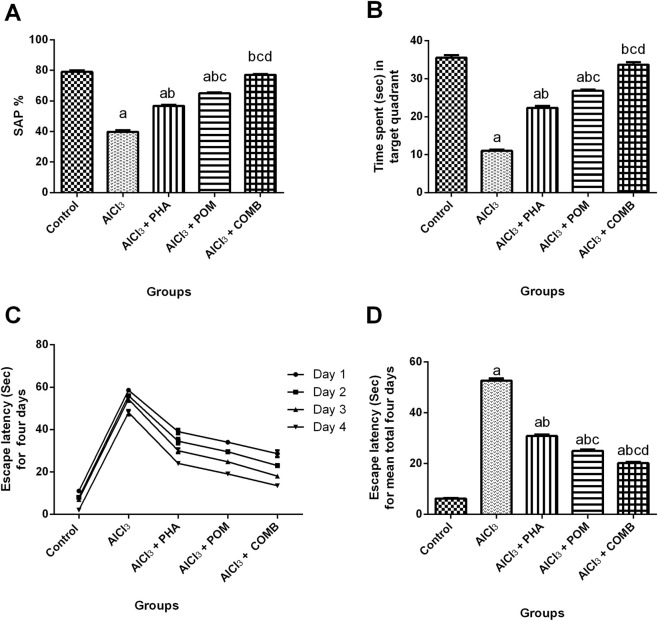
Effects of PHA and POM, either alone or in combination, on AlCl_3_-induced behavioral alterations in the Y-maze and MWM tests. SAP values in the Y-maze test **(A)**, time spent in the target quadrant in the MWM test **(B)**, escape latency time for 4 days in the MWM **(C)**, and escape latency time for a mean total of 4 days in the MWM **(D)**. Results are shown as the mean (n = 6) ± SEM. One-way ANOVA with post-test Tukey’s multiple comparison assessed the statistical differences between the various groups; *p*-value <0.05. Two-way ANOVA was used to analyze escape latency time for 4 days in the MWM. ^a^ a significant difference from control, ^b^ a significant difference from the AlCl_3_-intoxicated group, ^c^ a significant difference from the AlCl_3_ + PHA group, and ^d^ a significant difference from the AlCl_3_ + POM group. AlCl_3_, aluminum chloride; PHA, physical activity; POM, pomegranate extract; COMB, POM + PHA; SAP, spontaneous alteration percentage; MWM, Morris water maze.

As depicted in Figure 2, compared to the normal control group, AlCl_3_ markedly deteriorated spatial learning and memory functions, evidenced by the remarkable decrease in both SAP values in the Y-maze test and the time spent in the target quadrant in the MWM test (by 49.6% and 69%, respectively), along with the remarkable increment in the mean escape latency for the 4 days in the MWM test (8.5-fold) (Figures 2A–D). In contrast, PHA, POM, and their combination improved spatial learning and memory functions, verified by the marked increase in both SAP values in the Y-maze test (1.4-fold, 1.6-fold, and 1.9-fold, respectively), and time spent in the target quadrant in the MWM test (2-fold, 2.4-fold, and 3.1-fold, respectively), together with the pronounced decrease in the mean escape latency for the 4 days in the MWM test (by 41.5%, 52.5%, and 61.7%, respectively), relative to the AlCl_3_-intoxicated group. In this regard, PHA, POM, and their combination variably ameliorated AlCl_3_-induced behavioral alterations, moving toward normality, and the combination successfully restored both SAP values and the time spent in the target quadrant to their normal values.

#### 3.2.2 Effects of PHA and POM, either alone or in combination, on AlCl_3_-induced alterations in monoamine neurotransmitters (DA, NE, and 5-HT) and ACHE activity

**FIGURE 3:**
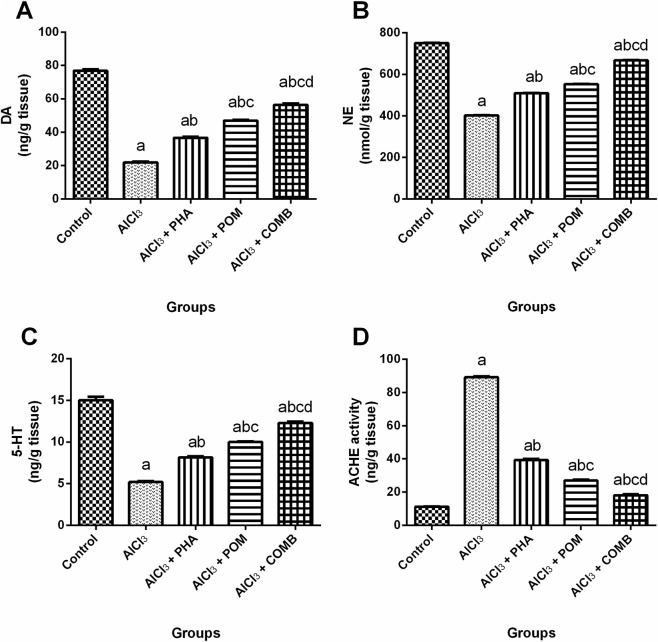
Effects of PHA and POM, either alone or in combination, on AlCl_3_-induced alterations in monoamine neurotransmitters and ACHE activity. Levels of DA **(A)**, NE **(B)**, 5-HT **(C)**, and ACHE activity **(D)**. Results are shown as the mean (n = 6) ± SEM. One-way ANOVA, along with post-test Tukey’s multiple comparison, assessed the statistical differences between the various groups; *p*-value <0.05. ^a^ a significant difference from control, ^b^ a significant difference from the AlCl_3_-intoxicated group, ^c^ a significant difference from the AlCl_3_ + PHA group, and ^d^ a significant difference from the AlCl_3_ + POM group. AlCl_3_, aluminum chloride; PHA, physical activity; POM, pomegranate extract; COMB, POM + PHA; DA, dopamine, NE, norepinephrine; 5-HT, serotonin; ACHE, acetylcholinesterase.

As shown in Figure 3, compared to the normal control group, AlCl_3_ substantially reduced monoamine neurotransmitter levels, including DA, NE, and 5-HT (by 71.4%, 46.4%, and 65.4%, respectively) (Figures 3A–C), whereas it increased ACHE activity (7.9-fold) (Figure 3D). Conversely, PHA, POM, and their combination markedly increased monoamine neurotransmitter levels, including DA (1.7-fold, 2.1-fold, and 2.6-fold, respectively), NE (1.3-fold, 1.4-fold, and 1.7-fold, respectively), and 5-HT (1.6-fold, 1.9-fold, and 2.4-fold, respectively), while they decreased ACHE activity (by 55.9%, 69.8%, and 79.7%, respectively), relative to the AlCl_3_-intoxicated group. In this context, PHA, POM, and their combination variably mitigated AlCl_3_-induced perturbations in monoamine neurotransmitters and ACHE activity, with movement, in the same sequence, toward normality.

#### 3.2.3 Effects of PHA and POM, either alone or in combination, on AlCl_3_-induced alterations in AD markers

**FIGURE 4:**
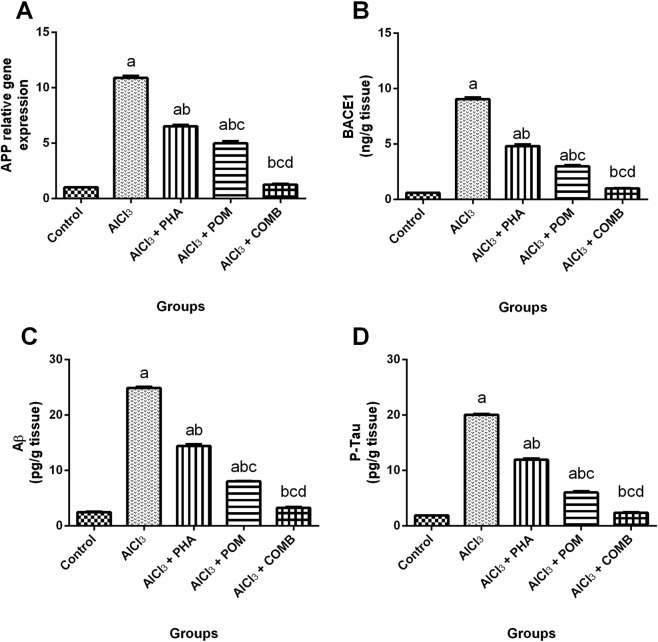
Effects of PHA and POM, either alone or in combination, on AlCl_3_-induced alterations in AD markers. mRNA expression level of APP **(A)** and levels of BACE1 **(B)**, Aβ **(C)**, and p-tau **(D)** in brain tissues. Results are shown as the mean (n = 6) ± SEM. One-way ANOVA, along with post-test Tukey’s multiple comparison, assessed the statistical differences between the various groups; *p*-value <0.05. ^a^ a significant difference from control, ^b^ a significant difference from the AlCl_3_-intoxicated group, ^c^ a significant difference from the AlCl_3_ + PHA group, and ^d^ a significant difference from the AlCl_3_ + POM group. AlCl_3_, aluminum chloride; PHA, physical activity; POM, pomegranate extract; COMB, POM + PHA; APP, amyloid precursor protein; BACE1, beta-secretase 1; Aβ, amyloid-beta; p-tau, phosphorylated-tau (hyperphosphorylated-tau).

As demonstrated in Figure 4, compared to the normal control group, AlCl_3_ elevated AD markers, including APP mRNA expression level, and BACE1, Aβ, and p-tau levels (10.8-fold, 15.3-fold, 10.1-fold, and 10.8-fold, respectively) (Figures 4A–C). In contrast, PHA, POM, and their combination reduced the mRNA expression level of APP (by 40.1%, 54.2%, and 88.3%, respectively), along with the levels of BACE1 (by 46.7%, 66.9%, and 89.1%, respectively), Aβ (by 42.1%, 67.8%, and 86.9%, respectively), and P-tau (by 40.6%, 70%, and 88.2%, respectively), relative to the AlCl_3_-intoxicated group. PHA, POM, and their combination alleviated AlCl_3_-induced alterations in AD markers, all moving, in the same order, toward normality, and the combination successfully restored the APP mRNA expression level, along with the BACE1, Aβ, and p-tau levels, to their normal values.

#### 3.2.4 Effects of PHA and POM, either alone or in combination, on AlCl_3_-induced alterations in APOE4 and neprilysin levels, together with the expression of LRP1

**FIGURE 5:**
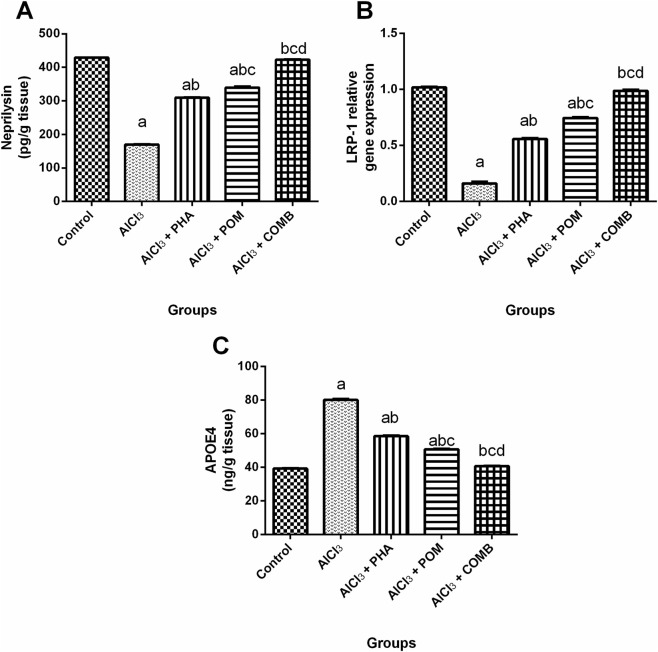
Effects of PHA and POM, either alone or in combination, on AlCl_3_-induced alterations in APOE4 and neprilysin levels, together with the expression of LRP1. The levels of neprilysin **(A)**, the mRNA expression level of LRP1 **(B)**, and the levels of APOE4 **(C)** in brain tissues. Results are shown as the mean (n = 6) ± SEM. One-way ANOVA, along with post-test Tukey’s multiple comparison, assessed the statistical differences between the various groups; *p*-value <0.05. ^a^ a significant difference from control, ^b^ a significant difference from the AlCl_3_-intoxicated group, ^c^ a significant difference from the AlCl_3_ + PHA group, and ^d^ a significant difference from the AlCl_3_ + POM group. AlCl_3_, aluminum chloride; PHA, physical activity; POM, pomegranate extract; COMB, POM + PHA; LRP1, low-density lipoprotein receptor-related protein 1; APOE4, apolipoprotein E4.

As shown in Figure 5, compared to the normal control group, AlCl_3_ diminished both the neprilysin level and the mRNA expression level of LRP1 (by 60.4% and 84.1%, respectively), whereas it markedly increased the APOE4 level (2-fold) (Figures 5A–C). In contrast, PHA, POM, and their combination elevated both neprilysin levels (1.8-fold, 2-fold, and 2.5-fold, respectively) and LRP1 mRNA expression levels (3.5-fold, 4.6-fold, and 6.1-fold, respectively), while they decreased APOE4 levels (by 26.9%, 36.7%, and 49.2%, respectively), relative to the AlCl_3_-intoxicated group. In this regard, PHA, POM, and their combination ameliorated AlCl_3_-induced alterations in the abovementioned parameters, moving toward normality, and the combination successfully restored the LRP1 mRNA expression level, along with the neprilysin and APOE4 levels, to their normal values.

#### 3.2.5 Effects of PHA and POM, either alone or in combination, on AlCl_3_-induced alterations in antioxidant and oxidative stress markers

**FIGURE 6:**
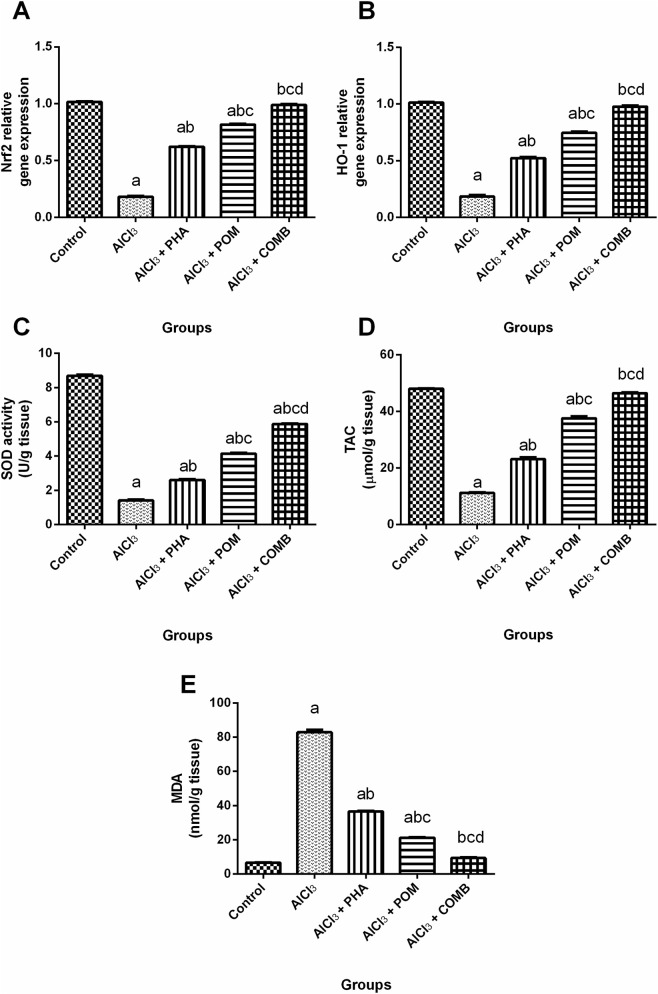
Effects of PHA and POM, either alone or in combination, on AlCl_3_-induced alterations in antioxidant and oxidative stress markers. mRNA expression levels of Nrf2 **(A)** and HO-1 **(B)** and the levels of SOD **(C)**, TAC **(D)**, and MDA **(E)** in brain tissue. Results are shown as the mean (n = 6) ± SEM. One-way ANOVA, along with post-test Tukey’s multiple comparison, assessed the statistical differences between the various groups; *p*-value <0.05. ^a^ a significant difference from control, ^b^ a significant difference from the AlCl_3_-intoxicated group, ^c^ a significant difference from the AlCl_3_ + PHA group, and ^d^ a significant difference from the AlCl_3_ + POM group. AlCl_3_, aluminum chloride; PHA, physical activity; POM, pomegranate extract; COMB, POM + PHA; Nrf2, nuclear factor-erythroid 2-related factor 2; HO-1, heme oxygenase-1; SOD, superoxide dismutase; TAC, total antioxidant capacity; MDA, malondialdehyde.

As shown in Figure 6, relative to the normal control group, AlCl_3_ produced vigorous oxidative stress, verified by the reduction in the mRNA expression levels of both Nrf2 and HO-1, and the levels of SOD and TAC (by 82.3%, 81.9%, 83.6%, and 76.6%, respectively) (Figures 6A–D), together with the increase in MDA level (12.4-fold) (Figure 6E). On the other side, PHA, POM, and their combination markedly increased the mRNA expression levels of both Nrf2 (3.4-fold, 4.5-fold, and 5.5-fold, respectively), and HO-1 (2.9-fold, 4.1-fold, and 5.3-fold, respectively), and the levels of SOD (1.8-fold, 2.9-fold, and 4.1-fold, respectively) and TAC (2.1-fold, 3.3-fold, and 4.1-fold, respectively), while they noticeably decreased MDA levels (by 55.8%, 74.4%, and 88.7%, respectively), compared to the AlCl_3_-intoxicated group. In this context, PHA, POM, and their combination mitigated AlCl_3_-induced perturbations in antioxidant and oxidative stress markers, moving toward normality, and the combination successfully restored the Nrf2 and HO-1 mRNA expression levels, along with the TAC and MDA levels, to their normal values.

#### 3.2.6 Effects of PHA and POM, either alone or in combination, on AlCl_3_-induced alterations in the expression of P38 MAPK/JNK/NF-κB and JAK-2/STAT-3 pathways, together with the levels of neuroinflammatory markers

**FIGURE 7:**
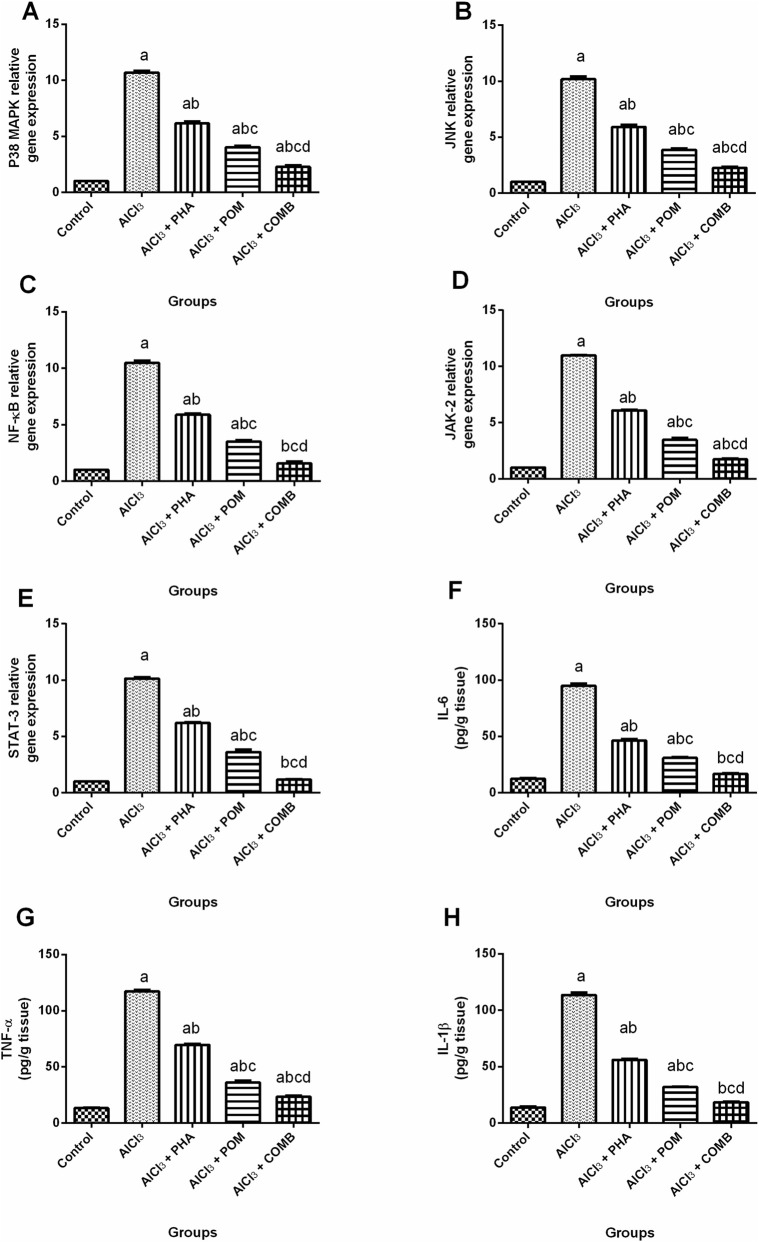
Effects of PHA and POM, either alone or in combination, on AlCl_3_-induced alterations in the expression of P38 MAPK/JNK/NF-κB and JAK-2/STAT-3 pathways, together with the levels of neuroinflammatory markers. mRNA expression levels of P38 MAPK **(A)**, JNK **(B)**, NF-κB **(C)**, JAK-2 **(D)**, and STAT-3 **(E)** and the levels of IL-6 **(F)**, TNF-α **(G)**, and IL-1β **(H)** in brain tissue. Results are shown as the mean (n = 6) ± SEM. One-way ANOVA, along with post-test Tukey’s multiple comparison, assessed the statistical differences between the various groups; *p*-value <0.05. ^a^ a significant difference from control, ^b^ a significant difference from the AlCl_3_-intoxicated group, ^c^ a significant difference from the AlCl_3_ + PHA group, and ^d^ a significant difference from the AlCl_3_ + POM group. AlCl_3,_ aluminum chloride; PHA, physical activity; POM, pomegranate extract; COMB, POM + PHA; P38 MAPK, P38 mitogen-activated protein kinase; JNK, c-Jun N-terminal kinase; NF-κB, nuclear factor-kappa beta; JAK-2, Janus kinase-2; STAT-3, signal transducer and activator of transcription 3; TNF-α, tumor necrosis factor-alpha; IL-1β, interleukin-1 beta; IL-6, interleukin-6.

As shown in Figure 7, compared to the normal control group, AlCl_3_ substantially increased the P38 MAPK, JNK, NF-κB, JAK-2 and STAT-3 mRNA expression levels (10.5-fold, 10-fold, 10.3-fold, 10.8-fold, and 9.9-fold, respectively) (Figures 7A–E), along with the levels of neuroinflammatory markers, including IL-6, TNF-α and IL-1β (7.7-fold, 8.8-fold, and 8.2-fold, respectively) (Figures 7F–H). PHA, POM, and their combination reduced the mRNA expression levels of P38 MAPK (by 42.4%, 62.5%, and 78.9%, respectively), JNK (by 41.8%, 62%, and 77.8%, respectively), NF-κB (by 43.9%, 66.6%, and 85.1%, respectively), JAK-2 (by 44.5%, 68.4%, and 84.2%, respectively), and STAT-3 (by 38.9%, 64.4%, and 88.4%, respectively), and the levels of IL-6 (by 51.2%, 67.4%, and 82.3%, respectively), TNF-α (by 40.6%, 69.1%, and 80%, respectively), and IL-1β (by 50.6%, 71.9%, and 83.8%, respectively), relative to the AlCl_3_-intoxicated group. In this way, PHA, POM, and their combination variably mitigated AlCl_3_-induced alterations in P38 MAPK/JNK/NF-κB hub, JAK-2/STAT-3 axis, and neuroinflammatory markers, moving toward normality, and the combination successfully restored NF-κB and the STAT-3 mRNA expression levels, along with the IL-6 and IL-1β levels, to their normal values.

#### 3.2.7 Effects of PHA and POM, either alone or in combination, on AlCl_3_-induced alterations in the expression of PI3K/AKT/GSK-3β/CREB/BDNF/TrKB hub

**FIGURE 8:**
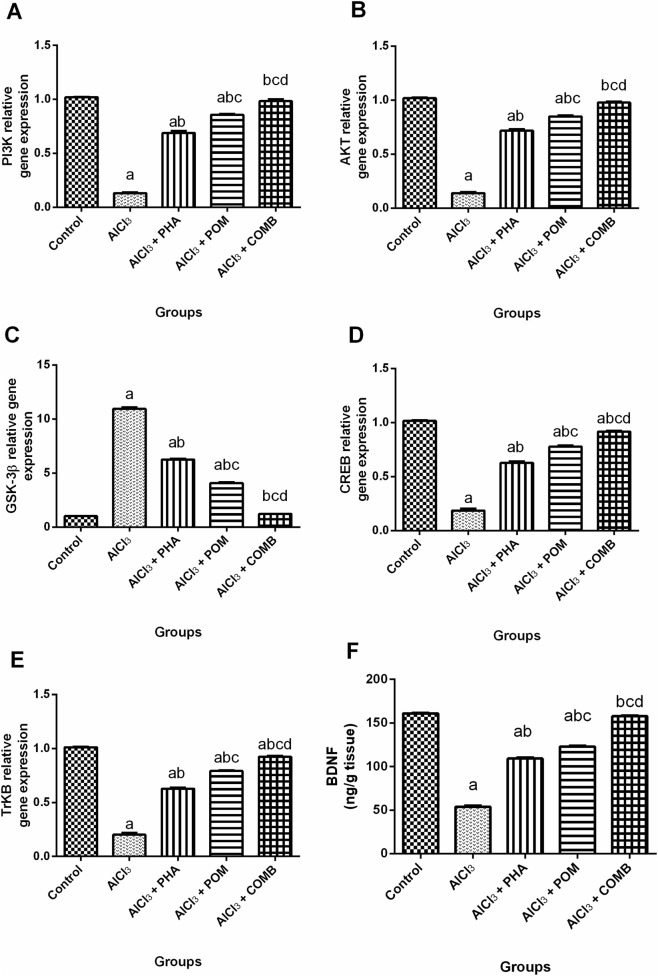
Effects of PHA and POM, either alone or in combination, either alone or in combination, on AlCl_3_-induced alterations in the expression of the PI3K/AKT/GSK-3β/CREB/BDNF/TrKB hub. mRNA expression levels of PI3K **(A)**, AKT **(B)**, GSK-3β **(C)**, CREB **(D)**, and TrKB **(E)** with the levels of BDNF **(F)** in brain tissue. Results are shown as the mean (n = 6) ± SEM. One-way ANOVA, along with post-test Tukey’s multiple comparison, assessed the statistical differences between the various groups; *p*-value <0.05. ^a^ a significant difference from control, ^b^ a significant difference from the AlCl_3_-intoxicated group, ^c^ a significant difference from the AlCl_3_ + PHA group, and ^d^ a significant difference from the AlCl_3_ + POM group. AlCl_3_, aluminum chloride; PHA, physical activity; POM, pomegranate extract; COMB, POM + PHA; PI3K, phosphoinositide 3-kinase; AKT, protein kinase B; GSK-3β, glycogen synthase kinase-3-beta; CREB, cyclic adenosine monophosphate (cAMP) response element-binding protein; TrKB, tropomyosin receptor kinase B; BDNF, brain-derived neurotrophic factor.

As shown in Figure 8, compared to the normal control group, AlCl_3_ noticeably reduced the PI3K, AKT, CREB, and TrKB mRNA expression levels, and the BDNF level (by 87.1%, 86.4%, 81.8%, 80.1%, and 66.6%, respectively) (Figures 8A,B,D–F), whereas it upregulated the mRNA expression level of GSK-3β (10.8-fold) (Figure 8C). In contrast, PHA, POM, and their combination substantially increased the mRNA expression levels of PI3K (5.2-fold, 6.5-fold, and 7.5-fold, respectively), AKT (5.2-fold, 6.1-fold, and 7.1-fold, respectively), CREB (3.4-fold, 4.2-fold, and 5-fold, respectively), and TrKB (3.1-fold, 3.9-fold, and 4.6-fold, respectively), and the levels of BDNF (2-fold, 2.3-fold, and 2.9-fold, respectively), while they downregulated the mRNA expression levels of GSK-3β (by 42.8%, 62.8%, and 88.9%, respectively), relative to the AlCl_3_-intoxicated group. In this regard, PHA, POM, and their combination variably alleviated AlCl_3_-induced dysregulation in the PI3K/AKT/GSK-3β/CREB/BDNF/TrKB axis, moving toward normality, and the combination successfully restored the PI3K, AKT, and GSK-3β mRNA expression levels, along with the BDNF level, to their normal values.

#### 3.2.8 Effects of PHA and POM, either alone or in combination, on AlCl_3_-induced alterations in ER stress and apoptotic markers

**FIGURE 9:**
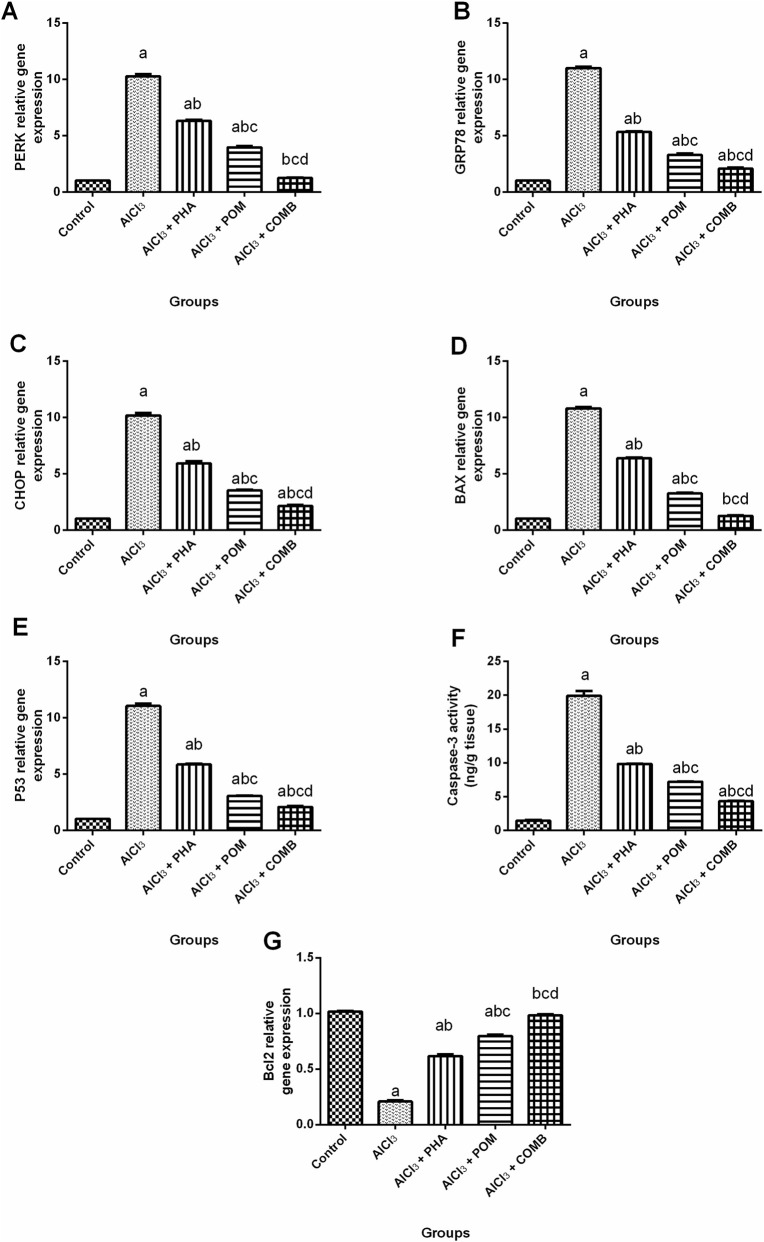
Effects of PHA and POM, either alone or in combination, on AlCl_3_-induced alterations in ER stress and apoptotic markers. mRNA expression levels of PERK **(A)**, GRP78 **(B)**, CHOP **(C)**, BAX **(D)**, P53 **(E)** and the levels of caspase-3 **(F)**, along with mRNA expression levels of Bcl-2 **(G)** in brain tissue. Results are shown as the mean (n = 6) ± SEM. One-way ANOVA, along with post-test Tukey’s multiple comparison, assessed the statistical differences between the various groups; *p*-value <0.05. ^a^ a significant difference from control, ^b^ a significant difference from the AlCl_3_-intoxicated group, ^c^ a significant difference from the AlCl_3_ + PHA group, and ^d^ a significant difference from the AlCl_3_ + POM group. AlCl_3_, aluminum chloride; PHA, physical activity; POM, pomegranate extract; COMB, POM + PHA; PERK, protein kinase RNA-like endoplasmic reticulum kinase; GRP78, glucose-regulated protein 78; CHOP, pro-apoptotic C/EBP homologous protein; Bcl-2, B-cell lymphoma-2 protein; BAX, B-cell lymphoma protein 2 (Bcl-2)-associated X protein.

As revealed in Figure 9, relative to the normal control group, AlCl_3_ increased both ER stress markers, including PERK, GRP78 and CHOP mRNA expression levels (10.1-fold, 10.8-fold, and 9.9-fold, respectively) (Figures 9A–C), and the pro-apoptotic markers, encompassing both the mRNA expression levels of BAX and P53 (10.7-fold, and 10.9-fold, respectively) (Figures 9D,E) and with the level of caspase-3 (13.8-fold) (Figure 9F). Simultaneously, AlCl_3_ downregulated the mRNA expression levels of the anti-apoptotic marker, Bcl-2 (by 79.5%) (Figure 9G), compared to the normal control group. Conversely, PHA, POM, and their combination decreased the mRNA expression levels of PERK (by 38.4%, 61.3%, and 87.8%, respectively), GRP78 (by 51.6%, 69.9%, and 81%, respectively), CHOP (by 41.8%, 65.4%, and 78.9%, respectively), BAX (by 40.9%, 69.9%, and 88.3%, respectively), and P53 (by 47%, 72.2%, and 81%, respectively), along with the levels of caspase-3 (by 50.6%, 63.7%, and 78.1%, respectively), relative to the AlCl_3_-intoxicated group. In parallel, PHA, POM, and their combination upregulated the mRNA expression levels of Bcl-2 (2.9-fold, 3.8-fold, and 4.7-fold, respectively), compared to the AlCl_3_-intoxicated group. In this way, PHA, POM, and their combination ameliorated AlCl_3_-induced alterations in ER stress and apoptotic markers, moving toward normality, and the combination successfully restored the PERK, BAX, and Bcl-2 mRNA expression levels to their normal values.

#### 3.2.9 Effects of PHA and POM, either alone or in combination, on AlCl_3_-induced alterations in the expression of the AMPK/SIRT-1 hub and autophagic markers

**FIGURE 10:**
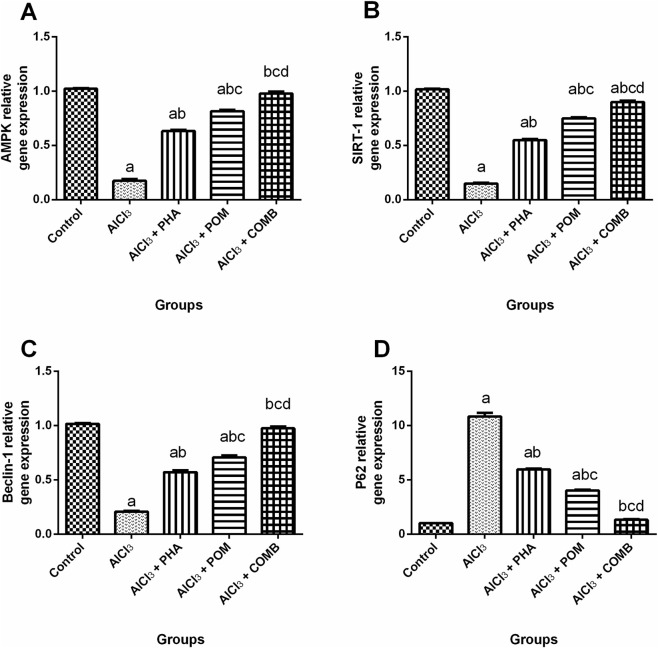
Effects of PHA and POM, either alone or in combination, on AlCl_3_-induced alterations in the expression of the AMPK/SIRT-1 hub and autophagic markers. mRNA expression levels of AMPK **(A)**, SIRT-1 **(B)**, Beclin-1 **(C)**, and P62 **(D)** in brain tissue. Results are shown as the mean (n = 6) ± SEM. One-way ANOVA, along with post-test Tukey’s multiple comparison, assessed the statistical differences between the various groups; *p*-value <0.05. ^a^ a significant difference from control, ^b^ a significant difference from the AlCl_3_-intoxicated group, ^c^ a significant difference from the AlCl_3_ + PHA group, and ^d^ a significant difference from the AlCl_3_ + POM group. AlCl_3_, aluminum chloride; PHA, physical activity; POM, pomegranate extract; COMB, POM + PHA; AMPK, adenosine monophosphate (AMP)-activated protein kinase; SIRT-1, sirtuin-1.

As shown in Figure 10, compared to the normal control group, AlCl_3_ downregulated the mRNA expression levels of AMPK, SIRT-1, and the autophagic marker, Beclin-1, (by 82.9%, 85.4%, and 79.7%, respectively) (Figures 10A–C), whereas it upregulated the mRNA expression level of the autophagic negative indicator, P62, (10.7-fold) (Figure 10D). In contrast, PHA, POM, and their combination upregulated the mRNA expression levels of AMPK (3.6-fold, 4.7-fold, and 5.6-fold, respectively), SIRT-1 (3.7-fold, 5-fold, and 6.1-fold, respectively), and Beclin-1 (2.8-fold, 3.4-fold, and 4.7-fold, respectively), while they downregulated the mRNA expression levels of P62 (by 44.9%, 62.9%, and 87.8%, respectively) relative to the AlCl_3_-intoxicated group. In this context, PHA, POM, and their combination alleviated AlCl_3_-induced changes in the AMPK/SIRT-1 axis and autophagic markers, moving toward normality, and the combination successfully restored the AMPK, Beclin-1, and P62 mRNA expression levels to their normal values.

#### 3.2.10 Effects of PHA and POM, either alone or in combination, on AlCl_3_-induced histopathological alterations in brain tissues

**FIGURE 11:**
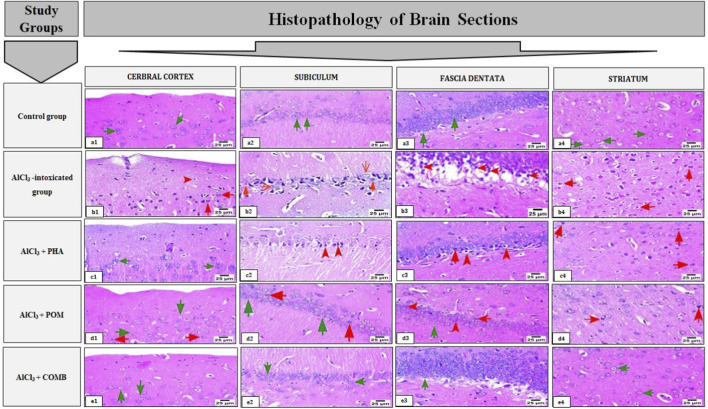
Effects of PHA and POM, either alone or in combination, on AlCl_3_-induced histopathological alterations in brain tissues. Represented photomicrographs of brain sections stained by hematoxylin and eosin (magnification ×40) of the control **(a1–a4)**, AlCl_3_ **(b1–b4)**, AlCl_3_ + PHA **(c1–c4)**, AlCl_3_ + POM **(d1–d4)**, and AlCl_3_ + COMB **(e1–e4)** groups, showing different histopathological alterations in the cerebral cortex, hippocampus (subiculum and fascia dentata) and striatum. AlCl_3_, aluminum chloride; PHA, physical activity; POM, pomegranate extract; COMB, POM + PHA. Keys: The green arrow indicates normal structure, the red arrow indicates necrosis, the red arrowhead indicates nuclear pyknosis, and the open red arrow indicates neuronal ferrugination.

As depicted in Figure 11, brain sections from the normal control group displayed well-preserved histological structure in the cerebral cortex, hippocampus (subiculum and fascia dentata), and striatum (green arrow) (Figures 11a1–11a4). In contrast, histopathological examination of the brain sections from the AlCl_3_-intoxicated group revealed massive eosinophilic necrosis (red arrow), accompanied by severe nuclear pyknosis and vacuolation of the neuropil (red arrowhead) in the cerebral cortex (Figure 11b1). Moreover, numerous severely necrotic neurons (red arrow) with hyperchromatic nuclei and neuronal ferrugination (open red arrow) are shown in the subiculum region of the hippocampus of the same group (Figure 11b2). A micrograph of the fascia dentata region of the hippocampus from the same group displayed severe neuronal necrosis (red arrow) (Figure 11b3). In addition, the striatum region of this group showed severe necrotic neurons (red arrow) (Figure 11b4).

These histopathological changes were variably ameliorated by PHA and POM, either alone or in combination. In the PHA group, an apparently normal structure of the cerebral cortex is displayed (Figure 11c1), while moderate necrosis (red arrow), moderate nuclear pyknosis (red arrowhead), and pericellular edema are shown in the subiculum and fascia dentata of the hippocampus from this group (Figures 11c2, 11c3). Additionally, the striatum region in the same group depicts mild necrotic neurons (red arrow) (Figure 11c4). In the POM group, histopathological examination of the cerebral cortex, subiculum, and fascia dentata exhibits almost normal neurons (green arrow), except for a few neurons that display mild eosinophilic necrosis (red arrow). Moreover, mild nuclear pyknosis (red arrowhead) is evident in all these regions (Figures 11d1–11d3). In addition, the striatum of this group shows mild nuclear pyknosis (red arrowhead) (Figure 11d4). Interestingly, the combination group exhibits mostly intact neuronal structures (green arrow) in the examined regions, including the cerebral cortex (Figure 11e1), the subiculum (Figure 11e2), and the fascia dentata (Figure 11e3) of the hippocampus, along with the striatum (Figure 11e4).

#### 3.2.11 Effects of PHA and POM, either alone or in combination, on AlCl_3_-induced alterations in GFAP immunoexpression in various brain regions

**FIGURE 12:**
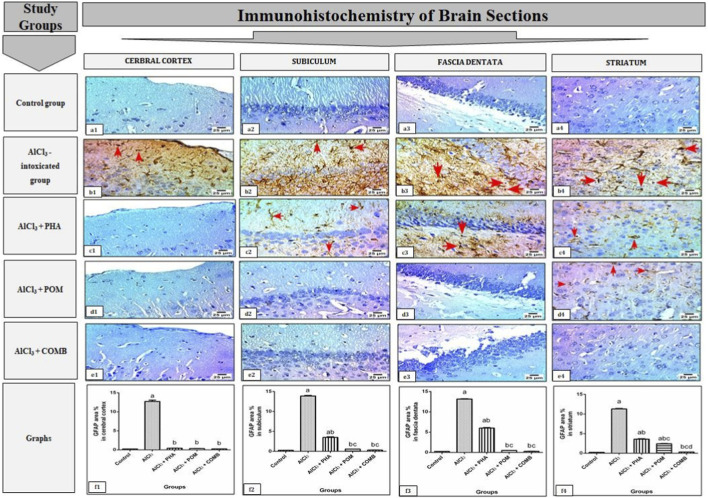
Effects of PHA and POM, either alone or in combination, on AlCl_3_-induced alterations in GFAP immunoexpression in various brain regions. Representative photomicrographs of immunohistochemical staining of GFAP-positive cells in various brain regions (red arrow) from different groups (magnification ×40). **(a)** Immunohistochemically stained brain sections from different areas in the control group showing negative immunoexpression for GFAP in the cerebral cortex **(a1)**, subiculum **(a2)**, and fascia dentata **(a3)** of the hippocampus and striatum **(a4)**. **(b)** Immunohistochemically stained brain sections from various areas in the AlCl_3_-intoxicated group showing intense GFAP immunoexpression (red arrow) in the cerebral cortex **(b1)**, subiculum **(b2)**, and fascia dentata **(b3)** of the hippocampus and the striatum **(b4)**. **(c)** Immunohistochemically stained brain sections from different areas in the AlCl_3_ + PHA group exhibiting negative GFAP immunoexpression in the cerebral cortex **(c1)**, while mild GFAP immunoexpression was detected in both the subiculum **(c2)** and the striatum **(c4)** (red arrow); moderate GFAP immunoexpression was demonstrated in the fascia dentata **(c3)** (red arrow). **(d)** Immunohistochemically stained brain sections from various regions in the AlCl_3_ + POM group displaying negative GFAP immunoexpression in the cerebral cortex **(d1)**, along with glial fibrillary acidic protein (GFAP) (1:800 dilution; Servicebio, Cat# GB12090) in the subiculum **(d2)** and fascia dentata **(d3)** of the hippocampus, whereas mild GFAP immunoexpression was noticed in the striatum **(d4)** (red arrow). **(e)** Immunohistochemically stained brain sections from different areas in the AlCl_3_ + COMB group, revealing negative immunoexpression for GFAP in all the examined brain areas, including the cerebral cortex **(e1)**, subiculum **(e2)**, and fascia dentata **(e3)** of the hippocampus and the striatum **(e4)**. **(f)** Bar charts representing the area % of GFAP staining for the studied groups in various brain areas, including the cerebral cortex **(f1)**, subiculum **(f2)**, and fascia dentata **(f3)** of the hippocampus and the striatum **(f4)**. Results are shown as the mean (n = 6) ± SEM. One-way ANOVA, along with post-test Tukey’s multiple comparison, assessed the statistical differences between the various groups; *p*-value <0.05. ^a^ a significant difference from control, ^b^ a significant difference from the AlCl_3_-intoxicated group, ^c^ a significant difference from the AlCl_3_ + PHA group, and ^d^ a significant difference from the AlCl_3_ + POM group. AlCl_3_, aluminum chloride; PHA, physical activity; POM, pomegranate extract; COMB, POM + PHA; GFAP, glial fibrillary acidic protein.

As shown in Figure 12, AlCl_3_ markedly increased GFAP immunoexpression in various brain regions, including the cerebral cortex, subiculum, and fascia dentata of the hippocampus, along with the striatum, compared to the normal control group. However, PHA, POM, and their combination lessened GFAP immunoexpression in the studied brain regions, by variable degrees, compared to the AlCl_3_-intoxicated group. First, in the cerebral cortex, AlCl_3_ increased GFAP immunoexpression (54-fold) (Figures 12b1, 12f1), relative to the normal control group. In contrast, PHA, POM, and their combination decreased GFAP immunoexpression in this area (by 96.5%, 97.3%, and 98%, respectively) (Figures 12c1, 12d1, 12e1, 12f1), compared to the AlCl_3_-intoxicated group. Second, in the subiculum of the hippocampus, AlCl_3_ increased GFAP immunoexpression (53.5-fold) (Figures 12b2, 12f2), relative to the normal control group. Conversely, PHA, POM, and their combination decreased GFAP immunoexpression in this region (by 74.8%, 96%, and 97.7%, respectively) (Figures 12c2, 12d2, 12e2, 12f2), compared to the AlCl_3_-intoxicated group. Third, in the fascia dentata of the hippocampus, AlCl_3_ increased GFAP immunoexpression (55.3-fold) (Figures 12b3, 12f3), relative to the normal control group. In contrast, PHA, POM, and their combination decreased GFAP immunoexpression in this area (by 54.3%, 96.4%, and 97.9%, respectively) (Figures 12c3, 12d3, 12e3, 12f3), compared to the AlCl_3_-intoxicated group. Finally, in the striatum, AlCl_3_ increased GFAP immunoexpression (49.6-fold) (Figures 12b4, 12f4), relative to the normal control group. On the other hand, PHA, vPOM, and their combination reduced GFAP immunoexpression in this region (by 68.3%, 78.9%, and 97.3%, respectively) (Figures 12c4, 12d4, 12e4, 12f4), compared to the AlCl_3_-intoxicated group.

Collectively, PHA, POM, and their combination variably alleviated AlCl_3_-induced alterations in GFAP immunoexpression in the studied brain regions, moving toward normality. In this regard, PHA successfully restored GFAP immunoexpression to its normal values in the cerebral cortex region only, while POM successfully restored GFAP immunoexpression to its normal values in the cerebral cortex and the subiculum and fascia dentata of the hippocampus. Interestingly, the combination exerted the maximum decrease in GFAP immunoexpression among the treated groups in all the studied brain regions, where it successfully restored GFAP immunoexpression to its normal values in all the investigated regions.

## 4 Discussion

Alzheimer’s disease (AD) is characterized by cognitive decline, hence negatively affecting patients’ quality of life (Silva et al., 2019). In fact, the predominance of Al in daily life activities makes exposure to it inescapable, thus increasing the risk for Al-associated neurotoxic consequences (Rather et al., 2019). The treatment of AD remains one of the major challenges (Liu et al., 2018). Therefore, searching for effective and safe neuroprotective strategies is a mainstay for the amelioration of AD. Among these approaches is the use of natural products with neuroprotective potentials, with safe profiles, ROS scavenging, and anti-inflammatory capabilities, making them a striking strategy for the mitigation of AD (Raj et al., 2021).

POM has been suggested to positively affect human health, in that it possesses antioxidant, anti-inflammatory, and neuroprotective actions (Doostan et al., 2017). Similarly, the role of PHA had been underscored in neurological maladies, where it boosts memory and cognitive functions (Lu et al., 2017). Given the fact that the pathophysiology of AD is complex and multifaceted, one of the chief tactics to ameliorate AD is the unearthing of a combination regimen that targets multiple pathways in mitigating AD (Huang and Mucke, 2012).

This study adopted a multimodal combinatorial regimen, involving the use of PHA, in conjunction with POM, a nutraceutical, as a promising approach that may possess favorable impacts against AD. Consequently, this study aimed to unravel and compare the neuroprotective effects of POM and PHA either alone or in combination against AlCl_3_-induced AD in rats, highlighting the role of the Nrf2/HO-1, P38 MAPK/JNK/NF-κB/JAK-2/STAT-3, PI3K/AKT/GSK-3β/CREB, AMPK/SIRT-1/Beclin-1, PERK/CHOP, amyloidogenic and apoptotic hubs as possible mechanistic contributors underlying their advantageous effects.

Compelling evidence suggests that AD is associated with marked perturbations in neurotransmitter levels, particularly monoaminergic and cholinergic neurotransmission systems, along with remarkable behavioral alterations that reflect memory impairment (Medhat et al., 2019; Vaz and Silvestre, 2020). Preserving normal monoaminergic and cholinergic neurotransmission systems is mandatory for normal brain functions, particularly memory functions (Yousefi Babadi et al., 2014; Azizi, 2022). In this regard, AlCl_3_ diminished monoamine neurotransmitter levels (DA, NE, and 5-HT) and increased ACHE activity, indicating lowered acetylcholine levels, which were coupled with alterations in spatial learning and memory functions, observed in both the Y-maze and MWM tests, compared to the normal control group. Such alterations in the behavioral outcomes, which implied that memory and cognitive functions were negatively affected by AlCl_3_, may be attributed to the disruption in neurotransmitter levels, including monoamines and acetylcholine, in this group.

These changes may be explained by the ability of aluminum to induce excessive ROS release and increase inflammatory response with consequent neuronal apoptosis and neuronal damage, where these malicious cascades were responsible for depleting neurotransmitter levels and its accompanying behavioral alterations (Awad et al., 2023; Salem et al., 2023). The alterations in neurochemical findings and behavioral outcomes, along with their accompanying neuronal damage in the AlCl_3_-intoxicated group, were corroborated by the histopathological findings that displayed structural disruption of the histopathological picture in various studied brain regions.

POM and PHA, either alone or in combination, ameliorated the alterations in monoamine neurotransmitter levels and ACHE activity, reflecting improvement in acetylcholine levels, along with the behavioral outcomes, to variable degrees, compared to the AlCl_3_-intoxicated group. These findings were further verified by the improvements in histopathological findings in all treated groups, to different extents, relative to the AlCl_3_-intoxicated group. The combination treatment produced more favorable actions than the standalone therapies. These positive impacts reflected the improvement in memory and cognitive functions exerted by the studied treatments, evinced by the improvement in behavioral findings, which may be attributed to the abilities of the tested agents to restore neurotransmitter levels, including monoamines and acetylcholine. In addition, these valuable actions may be attributed to the ability of PHA and the phenolic constituents of POM to exhibit neuroprotective, antioxidant, anti-inflammatory, and anti-apoptotic activities (Amri et al., 2017; de Almeida et al., 2022).

AD is marked by the excessive deposition of both Aβ, forming Aβ plaques (amyloidopathy), and p-tau, forming NFTs (tauopathy) (Khalifa et al., 2022). Notably, the excessive AD marker levels, APP, BACE1, Aβ, and p-tau, were reported to be closely connected with the incidence of increased oxidative stress and intensified inflammation (Li et al., 2012; Alawdi et al., 2017). In this context, AlCl_3_ increased APP expression, and BACE1, Aβ, p-tau, and APOE4 levels, while decreasing the LRP1 expression and neprilysin level, relative to the normal control group. The abovementioned increases in AD markers may be linked with the increase in oxidative stress and neuroinflammation in the AlCl_3_-intoxicated group. In addition, the alterations in AD markers may be correlated with the observed alterations in APOE4, neprilysin, and LRP1 levels in the AlCl_3_-intoxicated group. In this respect, the detected alterations in APOE4, neprilysin, and LRP1 levels in this group may be responsible for the incidence of imbalance between the production and degradation/clearance of AD markers, mainly Aβ and p-tau, which was strongly related to the elevation in AD markers (Khodadadi et al., 2018).

Regarding the increased production, the increased APOE4 levels were reported to be linked with promoted amyloidogenesis, through increasing the levels of APP, BACE1, and Aβ (Dafnis et al., 2018); these findings supported and clarified ours in the AlCl_3_-intoxicated group. Additionally, the increased APOE4 level was believed to be associated with the upregulation in GSK-3β and JNK expressions, as well as the increased p-tau level in the AlCl_3_-intoxicated group, where the heightened APOE4 levels were outlined to be largely linked with the promoted kinases, particularly GSK-3β and JNK, which contributed to the perturbed hyperphosphorylation of tau and the increased tauopathy (Ploia et al., 2011; Koutsodendris et al., 2022). While concerning the diminished degradation/clearance, the lessened neprilysin and LRP1 levels were found to be connected with the increased Aβ and p-tau levels in AD and hence the occurrence of amyloidopathy and tauopathy (Khalifa et al., 2022; Yang et al., 2025); these findings reinforced ours in the AlCl_3_-intoxicated group.

POM and PHA, either alone or in combination, alleviated the alterations in AD markers, APOE4 and neprilysin levels, and LRP1 expressions, to variable degrees, relative to the AlCl_3_-intoxicated group. The combination exerted more advantageous effects than solo therapy, outlining the possible augmentation between PHA and POM. These beneficial effects reflected the potential anti-Alzheimer activities of PHA and POM, where the decreased AD markers were associated with the documented antioxidant and anti-inflammatory activities exerted by PHA and the phenolic constituents of POM (Amri et al., 2017; de Almeida et al., 2022). Such amendments in AD markers by POM and PHA, either alone or in combination, may be connected with the improvements in APOE4, neprilysin, and LRP1 levels by these agents, which appeared to be responsible for the restoration in the balance between the production and degradation/clearance of AD markers, particularly Aβ and p-tau. In respect of the diminished production, the reduced APOE4 levels implied reduced amyloidopathy through the reduction of APP, BACE1, and Aβ in these groups. Moreover, the existing decreases in APOE4 levels were linked with the diminished expressions of both GSK-3β and JNK in these groups, reflecting inhibited tauopathy, evidenced by the reduced p-tau levels in these groups. However, concerning the increased degradation/clearance, the observed increases in neprilysin and LRP1 levels were accompanied by the reductions of both the Aβ and p-tau levels in these groups.

In addition to amyloidogenesis, dysregulations in redox, inflammatory, neurotrophic, ER stress, apoptotic, and autophagic machineries are also among the chief interconnected pathophysiological events involved in AD (Eshraghi et al., 2021; Awad et al., 2023; Si et al., 2023). Increased oxidative stress has been outlined as a key event in the etiopathology of AD, where excessive ROS levels induce neurodegeneration and neuronal apoptosis (Cheignon et al., 2018). In this frame, AlCl_3_ perturbed redox status, where it decreased Nrf2/HO-1 expression and antioxidant levels (TAC and SOD), while increasing the level of lipid peroxidation marker, MDA, versus the normal control group. These effects may be illustrated by the ability of aluminum to elicit surplus ROS release, resulting in the depletion of antioxidant defense machineries, including Nrf2, along with its downstream antioxidants, HO-1, SOD, and TAC (Kaur et al., 2021). Furthermore, these excessive ROS were reported to attack lipids, resulting in lipid peroxidation with the elevation in its marker, MDA (Cao et al., 2016). The brain is rich in PUFAs, making it highly susceptible to ROS-induced oxidative injury, with consequent lipid peroxidation, neurodegeneration, and neuronal apoptosis (Cobley et al., 2018). In addition, the increased GSK-3β expression has been reported to contribute to the suppression of Nrf2 signaling by promoting its degradation (Mousa et al., 2023). In this regard, aluminum-induced excessive ROS release and the upregulated GSK-3β expression in the AlCl_3_-intoxicated group acted together as possible contributors to the decrease in Nrf2 expression, with the consequent suppression in its downstream antioxidants, as well as the detected increase in lipid peroxidation in this group.

Conversely, POM and PHA either alone or in combination decreased the alterations in Nrf2/HO-1 expressions, with the levels of the antioxidants and the lipid peroxidation marker, by variable degrees, compared to those of the AlCl_3_-intoxicated group. The combination elicited more favorable impacts than solo therapy, delineating the possible augmentation between the advantageous effects of POM and PHA that acted to restore Nrf2/HO-1 signaling and increase its downstream antioxidants while repressing lipid peroxidation and thereby inhibiting neurodegeneration and neuronal apoptosis. These beneficial outcomes are largely linked to the antioxidant and anti-lipid peroxidative effects of POM and PHA (Amri et al., 2017; de Almeida et al., 2022). The phenolic constituents of POM, especially ellagic acid and punicalagin, were reported to scavenge ROS by acting as electron donors that react with free radicals to stabilize them, thus terminating free radical chain reaction and reducing lipid peroxidation (Tohame et al., 2010; Ibrahim et al., 2016). In addition, PHA and the phenolic constituents of POM were demonstrated to exert their antioxidant activities by acting as direct Nrf2 activators (Muthusamy et al., 2012; Akbarian et al., 2023). In this way, the ROS scavenging activities of POM, the direct Nrf2-activating abilities of POM and PHA, and the downregulated GSK-3β expressions in these groups may be responsible for the increase in the expression of Nrf2/HO-1 signaling, along with the resultant replenishment in the antioxidant defense and the decreased lipid peroxidation in these groups.

Ample evidence underscores the role of neuroinflammation in the pathophysiology of AD, where the excessive inflammatory response induces neurodegeneration and neuronal apoptosis (Javed et al., 2012). Indeed, the continued state of oxidative stress and neuroinflammation is a closely intertwined pathological event implicated in AD, as the increased oxidative stress contributes to heightened neuroinflammatory response (Horiguchi et al., 2018). One of the foremost cascades involved in neuroinflammation is the P38 MAPK/JNK/NF-κB hub, which plays a crucial part in the etiopathology of neurodegenerative maladies and is triggered by excessive ROS levels (Firdous et al., 2024).

In this context, AlCl_3_ exhibited an increased neuroinflammatory response, demonstrated by the increased P38 MAPK/JNK/NF-κB and JAK-2/STAT-3 expressions, along with the levels of pro-inflammatory mediators, including TNF-α, IL-1β, and IL-6, as well as GFAP immunoexpression, relative to the normal control group. This increase in the P38 MAPK/JNK axis may be ascribed to the ability of the AlCl_3_-induced excessive ROS release, which was associated with the downregulated Nrf2/HO-1 signaling, to instigate the P38 MAPK/JNK hub in this group. In addition, the increased expression of the P38 MAPK/JNK axis was reported to increase NF-κB expression, a fundamental controller of inflammatory response (Qi et al., 2019). In parallel, the increased GSK-3β expression was demonstrated to stimulate NF-κB signaling, via facilitating its nuclear translocation (Abu-Elfotuh et al., 2022). Upon initiation of NF-κB signaling, this resulted in the increased generation of its downstream pro-inflammatory mediators, encompassing TNF-α, IL-6, and IL-1β, which consequently promoted neurodegeneration and neuronal apoptosis (Yang et al., 2014; Kim et al., 2021). These data offered a probable illustration concerning the AlCl_3_-induced increase in NF-κB signaling, along with its downstream pro-inflammatory mediators, which may be attributed to the upregulated P38 MAPK/JNK and GSK-3β expressions in this group. The pro-inflammatory mediators, particularly IL-6, were documented to activate JAK-2/STAT-3 hub, another critical cascade implicated in intensification of neuroinflammatory response, resulting in subsequent astroglial hyperactivation, a hallmark of amplified neuroinflammation and neuronal injury (Jain et al., 2021). These data provided a plausible explanation for the AlCl_3_-induced upregulation in JAK-2/STAT-3 axis, where the AlCl_3_-induced increase in pro-inflammatory mediator levels, especially IL-6, may be responsible for the increase in JAK-2/STAT-3 expression, with the resultant astroglial overactivation, which was verified by the enhancement in GFAP immunoexpression in this group. Importantly, GFAP is produced by the activated astroglia, which produce ROS and pro-inflammatory mediators, thereby generating a vicious cycle between oxidative stress and neuroinflammation, promoting neurodegeneration (Colombo and Farina, 2016).

POM and PHA, either alone or in combination, inhibited the abovementioned chaos in the expressions of P38 MAPK/JNK/NF-κB and JAK-2/STAT-3 axes, together with the levels of pro-inflammatory mediators, to variable extents, compared to the AlCl_3_-intoxicated group. Interestingly, the combination exhibited more pronounced positive impacts than monotherapy, suggesting the possible enhancement between the beneficial actions of POM and PHA in inhibiting neuroinflammatory response. These effects may be related to the abilities of PHA, POM, and their combination to increase Nrf2 signaling with decreased ROS levels, inhibiting oxidative stress, which may lead to the observed downregulation in P38 MAPK/JNK expressions in these groups. Moreover, the observed downregulation in both P38 MAPK/JNK and GSK-3β cues in these groups may be responsible for the decrease in NF-κB expressions with its downstream pro-inflammatory mediators, and hence, decreasing neurodegeneration and neuronal apoptosis in these groups. In this frame, the suppression in the levels of pro-inflammatory mediators, particularly IL-6, by these agents may be responsible for the reduced JAK-2/STAT-3 expressions with the curtailing of astroglial activation, demonstrated by the diminished GFAP immunoexpression in these groups.

The dysregulation of the PI3K/AKT/GSK-3β/CREB/BDNF axis has been highlighted as a crucial pathological event in neurodegenerative maladies, including AD. In addition, the diminished CREB/TrKB/BDNF hub was outlined as a major contributor to the incidence of memory and cognitive impairments, along with decreased neuronal survival and increased neuronal apoptosis (Liu et al., 2022; Awad et al., 2023). In this regard, AlCl_3_ decreased PI3K/AKT/CREB/TrKB expressions and BDNF level, while it markedly upregulated GSK-3β expression, relative to the normal control group. The decrease in the CREB/TrKB/BDNF pathway provided clarification for the decline in memory and cognitive functions due to the increased apoptosis in the AlCl_3_-intoxicated group. The reduced AKT signaling, along with its accompanying increase in GSK-3β expression, was stated to promote oxidative stress, inflammation, and apoptosis, via curbing Nrf2 signaling and increasing both NF-κB and the apoptotic axes (Gouda and Cho, 2022; Mousa et al., 2023); these data supported ours and provided an illustration of the results of the AlCl_3_-intoxicated group.

POM and PHA, either alone or in combination, reduced the changes in PI3K/AKT/GSK-3β/CREB/BDNF hub, to variable extents, relative to the AlCl_3_-intoxicated group. The combination produced more favorable actions than solo therapy, highlighting the possible strengthening between the advantageous effects of POM and PHA in modulating the PI3K/AKT/GSK-3β/CREB/BDNF cue. At this point, the increased PI3K was stated to elicit phosphorylation of AKT, with subsequent activation of CREB and suppression of GSK-3β (Luo et al., 2021)—findings that supported ours. The activated CREB stimulated the expression of BDNF that interacted with TrKB to boost neuronal plasticity and neuronal survival, as well as cognitive and memory functions, while inhibiting neuronal apoptosis (Bagheri et al., 2021). These data align with ours and offer a possible explanation for the abilities of the tested agents to abolish both AlCl_3_-induced cognitive impairments and neuronal apoptosis in these groups. BDNF binding to TrKB also reactivated the PI3K/AKT hub to promote both CREB activation and the transcription of BDNF (Bathina and Das, 2015). Of note, the AKT-mediated suppression of GSK-3β in these groups may be responsible for the observed decrease in oxidative stress, neuroinflammatory response, and neuronal apoptosis in the same groups. Previous studies highlighted the ability of PHA and phenolic constituents of POM, particularly ellagic acid, to enhance memory functions via activation of BDNF signaling (Ridzwan et al., 2020; Alfaris et al., 2021; Cheng and Lee, 2022).

Another mechanistic event involved in the pathophysiology of neurodegenerative maladies, including AD, is the increased ER stress and its associated increased neuronal apoptosis (Ghemrawi and Khair, 2020; Awad et al., 2023). Notably, the increased oxidative stress and inflammatory response were found to be tightly correlated with the increased accumulation of misfolded proteins and the occurrence of increased ER stress (Hetz et al., 2020). In this context, AlCl_3_ upregulated the expressions of ER stress markers, PERK/GRP78/CHOP, relative to the normal control group. At this point, the increase in ER stress by AlCl_3_ may be related to the increased oxidative stress and neuroinflammation in the same group. Of note, the increased ER stress may be connected with the increased Aβ and p-tau levels, misfolded proteins, and the incidence of proteinopathy in the same group. The occurrence of increased ER stress resulted in overstimulation of UPR and the activation of PERK, leading to an increase in CHOP, a pro-apoptotic factor, which initiated apoptotic signaling and inhibited the anti-apoptotic Bcl-2 (Romine and Wiseman, 2019). In addition to the ER stress, oxidative stress, neuroinflammation, and elevated GSK-3β expression with the reduced AKT expression were also reported to act as inducers to neuronal apoptosis (Wang et al., 2007; Redza-Dutordoir and Averill-Bates, 2016). The decreased AKT signaling was tightly associated with the increased apoptosis, through P53 elevation and apoptotic signaling (Awad et al., 2023). Based upon these findings, it can be anticipated that the increase in ER stress markers, particularly CHOP, acted together with the existing ROS, neuroinflammatory mediators, and GSK-3β, along with the decreased AKT expression, to induce apoptotic events in the AlCl_3_-intoxicated group. This outcome is shown by the elevation in the apoptotic markers (BAX/P53 expressions and caspase-3 level) and the downregulation in anti-apoptotic Bcl-2 expression in this group, compared to the normal control group.

POM and PHA, either alone or in combination, decreased both ER stress markers (PERK/GRP78/CHOP) and apoptotic markers (BAX, P53, and caspase-3), while increasing the anti-apoptotic marker, Bcl-2, by different amounts relative to the AlCl_3_-intoxicated group. The combination elicited more positive impacts than the monotherapy, outlining the possible reinforcement between the favorable effects of POM and PHA in inhibiting ER stress and neuronal apoptosis. In this regard, the reduction of ER stress by POM, PHA, and their combination may be linked with the antioxidant and anti-inflammatory effects of these agents. Additionally, the reduction of ER stress by these agents may also be correlated with the detected diminished Aβ and p-tau levels, and thereby curtailed proteinopathy in these groups. In this way, the decreased ER stress, oxidative stress, neuroinflammation, and GSK-3β expressions, together with the observed increase in AKT expressions, via acting cooperatively, may be responsible for the inhibition of neuronal apoptosis in these groups. Previous studies underscored the ability of PHA and phenolic constituents of POM to inhibit ER stress (Kang et al., 2013; Mansour et al., 2025).

Diminished autophagy is a crucial pathophysiological mechanism implicated in neurodegenerative illness, including AD (Wang and Jia, 2023). Autophagy is responsible for the clearance of misfolded proteins, thus preserving proteostasis, increasing neuronal survival, and reducing apoptosis. At this point, the decreased autophagy was outlined to be strongly related to the presence of amplified deposition of misfolded proteins, increased ER stress, and its associated increased neuronal apoptosis (Fowler and Moussa, 2018). Additionally, AMPK/SIRT-1 cue, a pivotal axis for regulating autophagy, is disrupted in neurodegenerative maladies (Ibrahim et al., 2022). In this regard, AlCl_3_ abrogated autophagy, evidenced by the downregulation in the expressions of AMPK/SIRT-1 and the autophagic marker, Beclin-1, along with upregulation in the autophagic negative indicator, P62 expression, in this group, relative to the normal control group. The impaired autophagy in the AlCl_3_-intoxicated group may be correlated with the observed increase in Aβ and p-tau levels, proteinopathy, the detected increased ER stress, and its accompanying increased neuronal apoptosis, along with cognitive decline and neurodegeneration in the same group.

In contrast, POM and PHA either alone or in combination noticeably boosted autophagy, as shown by the upregulation in the expressions of AMPK/SIRT-1/Beclin-1, together with downregulation in the expression of P62, by different degrees, in these groups, compared to the AlCl_3_-intoxicated group. The combination produced more favorable impacts than the standalone therapy, reflecting the possible strengthening between the beneficial actions of POM and PHA in increasing autophagy with modulation of the AMPK/SIRT-1 axis. In this way, the activated AMPK was reported to activate SIRT-1, thereby increasing autophagy, promoting the expression of autophagy-related marker, Beclin-1, while inhibiting the expression of the autophagic negative indicator, P62, along with increasing the clearance of the accumulated misfolded proteins, diminishing ER stress, decreasing neuronal apoptosis, and improving neuronal survival (Qu et al., 2019; Deng et al., 2020). These findings support ours in these groups. In this context, the autophagic-increasing capabilities of POM, PHA, and their combination may be connected with the observed decrease in Aβ and p-tau levels, reduced proteinopathy, restored proteostasis, decreased ER stress, enhanced neuronal survival, improvement in cognitive functions, and neuroprotection in these groups. Several studies documented that PHA and phenolic constituents of POM increased autophagic machineries (Brandt et al., 2018; Tan et al., 2019).

## 5 Conclusion

This study revealed the critical role of the dysregulations in the Nrf2/HO-1, P38 MAPK/JNK/NF-κB, JAK-2/STAT-3, PI3K/AKT/GSK-3β/CREB, AMPK/SIRT-1, PERK/CHOP, amyloidogenic and apoptotic hubs, as key pathological mechanisms, in AlCl_3_-provoked neurotoxicity. Our results show that POM and PHA, either alone or in combination, mitigated AlCl_3_-induced tauo-/amyloidopathy, where they ameliorated its associated cognitive dysfunction, along with the dysregulations in behavioral outcomes, neurotransmitter levels, redox, neuroinflammatory, neurotrophic, ER stress, autophagic, amyloidogenic, and apoptotic machineries, along with its related histopathological and immunohistochemical alterations, by variable grades. These favorable impacts of the tested agents were exerted via modulation of the Nrf2/HO-1, P38 MAPK/JNK/NF-κB, JAK-2/STAT-3, PI3K/AKT/GSK-3β/CREB, AMPK/SIRT-1, PERK/CHOP, and amyloidogenic and apoptotic axes. The combination of PHA with nutraceutical POM exhibited more favorable impacts in all the assessed behavioral outcomes, neurotransmitter levels, biochemical parameters, together with histopathological and immunohistochemical findings, than those exerted by either standalone therapy. These findings delineate the possible augmentation between the advantageous actions of POM and PHA in boosting cognitive functions, redox mechanisms, neurotrophic and autophagic machineries while inhibiting neuroinflammation, ER stress, and apoptotic cascades in AlCl_3_-induced AD. Accordingly, our findings revealed the beneficial effects of integrating the use of PHA in conjunction with POM as a promising multimodal combinatorial regimen in the amelioration of AD.

## 6 Future recommendations

1. The current findings of our study provided emerging pre-clinical evidence regarding the use of this combinatorial approach as a promising avenue in the mitigation of AD. Accordingly, it is recommended that clinical studies be conducted using this combinatorial approach to emphasize and ensure both its clinical effectiveness and its translational potential in human populations with AD.
2. Future studies should use tracking software such as ANY-maze to benefit from assessing numerous behavioral variables within the behavioral test, which provides more comprehensive behavioral assessment for the effects of various therapeutic interventions on the neurological functions in experimental animal models.
3. Future studies should incorporate more behavioral tests, such as the novel object recognition test, to gather more clues about the impacts of different therapeutic interventions on the neurological functions in experimental animal models.

## Data Availability

The original contributions presented in the study are included in the article/supplementary material; further inquiries can be directed to the corresponding author.
